# The effect and safety of Tai Chi on bone health in postmenopausal women: A meta-analysis and trial sequential analysis

**DOI:** 10.3389/fnagi.2022.935326

**Published:** 2022-09-13

**Authors:** Xiaobo Liu, Chengzhi Jiang, Rong Fan, Tianyu Liu, Yuxi Li, Dongling Zhong, Luxiang Zhou, Tao Liu, Juan Li, Rongjiang Jin

**Affiliations:** ^1^School of Health Preservation and Rehabilitation, Chengdu University of Traditional Chinese Medicine, Chengdu, China; ^2^Department of Rehabilitation Medicine, Sichuan Science City Hospital, Mianyang, China; ^3^Department of Rehabilitation Medicine, Nanbu County People's Hospital, Nanchong, China; ^4^School of Sport and Health, Chengdu University of Traditional Chinese Medicine, Chengdu, China; ^5^Integrated Traditional Chinese and Western Medicine Hospital of Panzhihua City, Panzhihua, China

**Keywords:** Tai Chi, post-menopause, BMD, meta-analysis, trial sequential analysis

## Abstract

**Background:**

Tai Chi may be a promising exercise to prevent and control bone loss in postmenopausal women. This meta-analysis and trial sequential analysis aimed to evaluate the effect and safety of Tai Chi on bone health in postmenopausal women.

**Method:**

Seven databases were searched from their inceptions to 11 May 2022 to collect randomized controlled trials (RCTs) investigating the effect and safety of Tai Chi on bone health in postmenopausal women. Two independent reviewers identified the eligible studies, extracted data, and assessed the risk of bias of included studies using the revised Cochrane risk-of-bias tool for randomized trials. The primary outcome was the bone mineral density (BMD), and secondary outcomes included bone turnover markers and calcaneus quantitative ultrasound. Subgroup analyses were conducted based on the duration of Tai Chi. Sensitivity analyses and publication bias assessment were performed. RevMan software (version 5.4.1) and R software (version 3.6.1) were used for data synthesis. The certainty of evidence was rated with the Grading of recommendations assessment, development, and evaluation (GRADE) system. We also performed the trial sequential analysis to evaluate the reliability of the evidence.

**Results:**

A total of 25 reports involving 24 studies were included. Four studies were considered as high overall risk of bias, and the rest were some concerns. Among included studies, there were three comparisons including Tai Chi vs. non-intervention, Tai Chi vs. other exercises, and Tai Chi plus nutraceutical vs. nutraceutical. Compared with non-intervention, Tai Chi was more effective to improve BMD of lumbar spine (MD = 0.04, 95% CI 0.02 to 0.07, *I*^2^ = 0%, low certainty), femoral neck (MD = 0.04, 95% CI 0.02 to 0.06, *I*^2^ = 0%, low certainty), and trochanter (MD = 0.02, 95% CI 0.00 to 0.03, *I*^2^ = 0%, very low certainty), but there was no significant difference in increasing the BMD of Ward's triangle (MD = 0.02, 95% CI −0.01 to 0.04, *I*^2^ = 0%, very low certainty). Trial sequential analysis showed that the effect of Tai Chi vs. non-intervention on the BMD of lumbar spine and femoral neck was reliable, but the effect on the BMD of trochanter and Ward's triangle needed further verification. The subgroup analyses suggested that Tai Chi training for over 6 months had greater improvement in BMD of the lumbar spine, femoral neck, and trochanter than non-intervention. No significant differences were observed in the above outcomes of Tai Chi vs. other exercises, and Tai Chi plus nutraceutical vs. nutraceutical. There was insufficient evidence to support the effect of Tai Chi on bone turnover markers and calcaneus quantitative ultrasound. Few Tai Chi relevant adverse events occurred.

**Conclusion:**

Tai Chi may be an optional and safe exercise for improving BMD loss in postmenopausal women, and practicing Tai Chi for more than 6 months may yield greater benefits. However, more rigorously designed RCTs are required to verify the benefits and to explore the optimal protocol of Tai Chi exercise for bone health.

**Systematic review registration:**

https://www.crd.york.ac.uk/prospero/display_record.php?RecordID=309148, identifier: CRD42022309148.

## Introduction

Post-menopause is a period of women's life following the permanent cessation of the menstrual cycles, during which time the women's bone health is threatened due to the decrease and cessation of ovarian estrogen secretion (Cauley, 2015). Bone mineral density (BMD) decreases rapidly during the menopause transition and continues to decline in post-menopause. According to a cohort study involving 1,038 women (Shieh et al., 2022), each additional year after the final menstrual period was associated with 0.006 and 0.004 g/cm^2^ lower BMD of lumbar spine and femoral neck, respectively. The 10-year cumulative loss of BMD was 10.6% at the lumbar spine and 9.1% at the femoral neck (Cauley, 2015). Low BMD was one of the most important determinants of fracture risk (Barron et al., 2020). Approximately 30–40% of postmenopausal women were reported to have osteoporosis or low bone mass (Wright et al., 2014; Thulkar et al., 2016), and more than 30% experienced at least one fracture (Lippuner et al., 2009; Si et al., 2015; Jiang and Ni, 2016). Almost every fracture was associated with an increased risk of premature mortality (Center, 2017). Researchers found mortality increased over 2.43- and 1.82-fold following hip fractures and vertebral fractures in community-dwelling older women, respectively (Bliuc et al., 2009). Therefore, effective intervention to prevent and attenuate bone loss in postmenopausal women is necessary.

Bone is a dynamic tissue, with a capacity to remodel its material and structural properties to adapt mechanical loading (Feng and McDonald, 2011). Increased loading stimuli and vigorous muscular activity can augment bone mass and promote bone health (Wang et al., 2020). Therefore, exercise is recommended to maintain bone mass or slow bone loss for postmenopausal women (Daly et al., 2019; Kanis et al., 2019; Society, 2021). High impact and high weight-bearing exercises were found to be beneficial for postmenopausal women to increase BMD (Martyn-St James and Carroll, 2006; Kelley et al., 2012; Zhao et al., 2015; Kitsuda et al., 2021). However, due to safety and operability concerns, it's difficult to implement such exercise patterns for post-menopause women.

Tai Chi, a traditional Chinese exercise, is becoming popular around the world. According to the theory of traditional Chinese medicine, Tai Chi can promote the circulation of Qi and blood. Tai Chi is characterized by coordinated body posture and movements, deep rhythmic breathing, and meditation (Yeung et al., 2018). During Tai Chi practice, practitioners perform a series of slow and rhythmic circular motions and a lot of half-squats, and gravity-shift movements, which may introduce dynamic loading on bone. Evidence showed Tai Chi can prevent falls, enhance flexibility and improve balance function with good security (Del-Pino-Casado et al., 2016; Zhong et al., 2020). In addition, it is an easily acceptable exercise that can be practiced anywhere and anytime without special equipment. Therefore, Tai Chi may be a promising exercise to prevent and reduce bone loss in postmenopausal women.

Recently, Zhang et al. (2021) conducted a network meta-analysis and found mind-body exercise (e.g. Tai Chi, yoga, dance, Wuqinxi) might be an optimal exercise type to increase the BMD of the lumbar spine and femoral neck among patients with osteoporosis and osteopenia. Previous two systematic reviews (SRs) of Tai Chi for BMD in postmenopausal women had been published in 2016 (Sun et al., 2016) and 2017 (Liu and Wang, 2017) respectively, but their conclusions were contradictory. As more relevant trials have been conducted in recent years, we performed this meta-analysis of randomized controlled trials (RCTs) to update the evidence about the effect and safety of Tai Chi on bone health in postmenopausal women and used the trial sequential analysis (TSA) to assess the reliability of the evidence.

## Materials and methods

### Study registration

The protocol of this meta-analysis and TSA has been registered on the International Prospective Register of Systematic Reviews (PROSPERO) (https://www.crd.york.ac.uk/prospero/display_record.php?RecordID=309148) (Registration No: CRD42022309148). We conducted this meta-analysis and TSA according to A Measurement Tool to Assess Systematic Reviews (AMSTAR 2) and reported in the light of the Preferred Reporting Items for Systematic Review and Meta-Analysis (PRISMA) 2020 statement (Appendix 1).

### Search strategy

Reviewers (YXL and DLZ) searched PubMed, Embase, The Cochrane Library, China National Knowledge Infrastructure (CNKI), Chinese Science and Technology Periodical Database (VIP), Chinese Biomedical Literature Database (CBM), and Wanfang Database from their inceptions to 11 May 2022. According to the retrieval rules of each database, the search strategies were developed by combining Medical Subject Headings (MeSH) and free text words of Tai Chi and bone density. To identify more potential studies, we manually searched gray literature, reference lists of identified studies, and relevant registration websites (ClinicalTrials.gov and www.chictr.org.cn), and consulted experts in this field. The full search strategies for all databases are shown in Appendix 2.

### Inclusion criteria

#### Type of studies

We included RCTs published in Chinese and English which studied the effect or/and safety of Tai Chi on bone health in postmenopausal women.

#### Type of participants

Postmenopausal women (author reported) or women (≥ 50 years old) (Wang et al., 2021) were included. There was no restriction on race or nation.

#### Type of interventions

We included RCTs that used Tai Chi (e.g. Tai Chi Quan, Tai Chi push hands, Tai Chi sword, etc.), or Tai Chi combined anti-osteoporosis medications (e.g. bisphosphonates, denosumab, calcitonin, etc.) or nutraceutical (e.g. calcium and vitamin D, etc.) as the experimental group. There were no restrictions on the duration and frequency of Tai Chi.

#### Type of comparators

Participants in the control group received non-intervention, anti-osteoporosis drug, nutraceutical, or other exercises (e.g. walking, running, resistance training, etc.).

#### Outcome measurements

The primary outcome included BMD using dual-energy x-ray absorptiometry (lumbar spine, femoral neck, trochanter, Ward's triangle, and total hip). Secondary outcomes were indicators related to bone health, including: 1) Calcaneus quantitative ultrasound: BMD of the calcaneus, bone quality index, broadband ultrasound attenuation, speed of sound; 2) Bone turnover markers: serum bone formation markers: procollagen type I N-terminal propeptide (PINP), alkaline phosphatase (ALP), bone-specific alkaline phosphatase (BAP), osteocalcin (OSC), etc.; serum bone resorption markers: C-terminal telopeptide of type I collagen (CTX), tartrate-resistant acid phosphatase (TRAP), etc.; 3) Tai Chi-related adverse events.

### Exclusion criteria

Studies were excluded if they met any of the following conditions: 1) Cross-sectional studies, reviews, case-control studies, N of one RCTs (Li et al., 2022); 2) Full text or the data cannot be obtained through various approaches; 3) Repeated publications.

### Study selection

All the retrieved records were imported into Endnote software (X9), and duplicates were removed. Two independent reviewers (LXZ and TL) screened the rest records by reading titles and abstracts. Then, full texts of all potential studies were obtained and scrutinized. After that, the two reviewers cross-checked the included studies. In case of disagreements, a third reviewer (JL) was involved. If there were multiple publications from the same study, we included the publication with more complete data or included multiple publications with complementary data.

### Data extraction

Two reviewers (CZJ and RF) independently extracted the following data: 1) Study characteristics: first author, publication year, country, sample size; 2) Participants' characteristics at study level: age, menopausal duration; 3) Interventions: frequency, duration, and style of Tai Chi; 4) Comparators: type, dosage, frequency and duration of medication or nutraceutical; frequency, duration, type of other exercises; 5) Outcomes: primary outcome, secondary outcomes, and adverse events; 6) Information related to the risk of bias. Then two reviewers cross-checked the extracted data to ensure no mistakes. We resolved discrepancies through group discussion or with the participation of a third reviewer (JL). For multi-arm RCTs, we included the eligible comparisons or extracted the comparison with inferior effect size to obtain more conservative results.

We contacted the original authors *via* email for more information if the necessary data was missing or incomplete. If there was no reply, we analyzed the available data.

### Risk of bias assessment

Two independent reviewers (XBL and TYL) used the revised Cochrane risk-of-bias tool for randomized trials (ROB 2) to assess the risk of bias of included studies from five domains: the randomization process, deviations from intended interventions, missing outcome data, measurement of the outcome, and selection of the reported result. Each domain was judged as “low risk,” “some concerns,” or “high risk” according to corresponding algorithms. After learning the Cochrane risk-of-bias tool and pre-assessed, two independent reviewers assessed the risk of bias and then cross-checked. Two reviewers discussed the disagreements or consulted with a third reviewer (RJJ).

### Data analysis

Among the included studies, three comparisons were involved, including Tai Chi vs. non-intervention, Tai Chi vs. other exercises, and Tai Chi plus nutraceutical vs. nutraceutical. For continuous variable, we used the post-intervention data. Since included outcomes for meta-analysis used the same units, we calculated the mean difference (MD). We conducted descriptive analysis for the data which couldn't be quantitatively analyzed. Heterogeneity was measured by the chi-squared test and *I*^2^ statistic. When *P* < 0.1 or *I*^2^ values > 50%, the random-effect model was used to pool data. Otherwise, the fixed-effect model was performed. Forest plots and tables were utilized to present the pooled results. RevMan software (version 5.4.1) and R software (version 3.6.1) were used for data synthesis.

### Subgroup analysis

Subgroup analyses of Tai Chi vs. non-intervention were conducted according to the duration of Tai Chi ( ≤ 6 or > 6 months).

### Sensitivity analysis

We performed sensitivity analysis by eliminating studies one by one to verify the robustness of the results.

### Publication bias

We used a funnel plot and *Egger's* test to detect publication bias when ≥10 studies with the same outcome were included in the analysis.

### TSA

We conducted TSA for primary outcome using the TSA software (version 0.9.5.10-Beta). Fixed effects model with a maximum type I error of 5%, and a maximum type II error of 20% (80% power) were applied. Two-sided significance testing boundaries, required information size, trial sequential monitoring boundaries, futility boundaries, and cumulative z-score were presented in the TSA graph. The situation that included sample size over required information size, or the cumulative Z curve crossed the trial sequential monitoring boundaries or futility boundaries indicated that the results were reliable.

### Certainty of evidence

We applied the Grading of recommendations assessment, development, and evaluation (GRADE) system to assess the certainty of evidence. Each outcome was evaluated from the following five aspects: limitations, inconsistency, indirectness, imprecision, and publication bias. Then the certainty of evidence was accordingly graded as “high,” “moderate,” “low,” or “very low” (Balshem et al., 2011). GRADEpro (version 3.6) software was used to present the summary of findings.

## Results

### Study inclusion and characteristics

**Figure 1:**
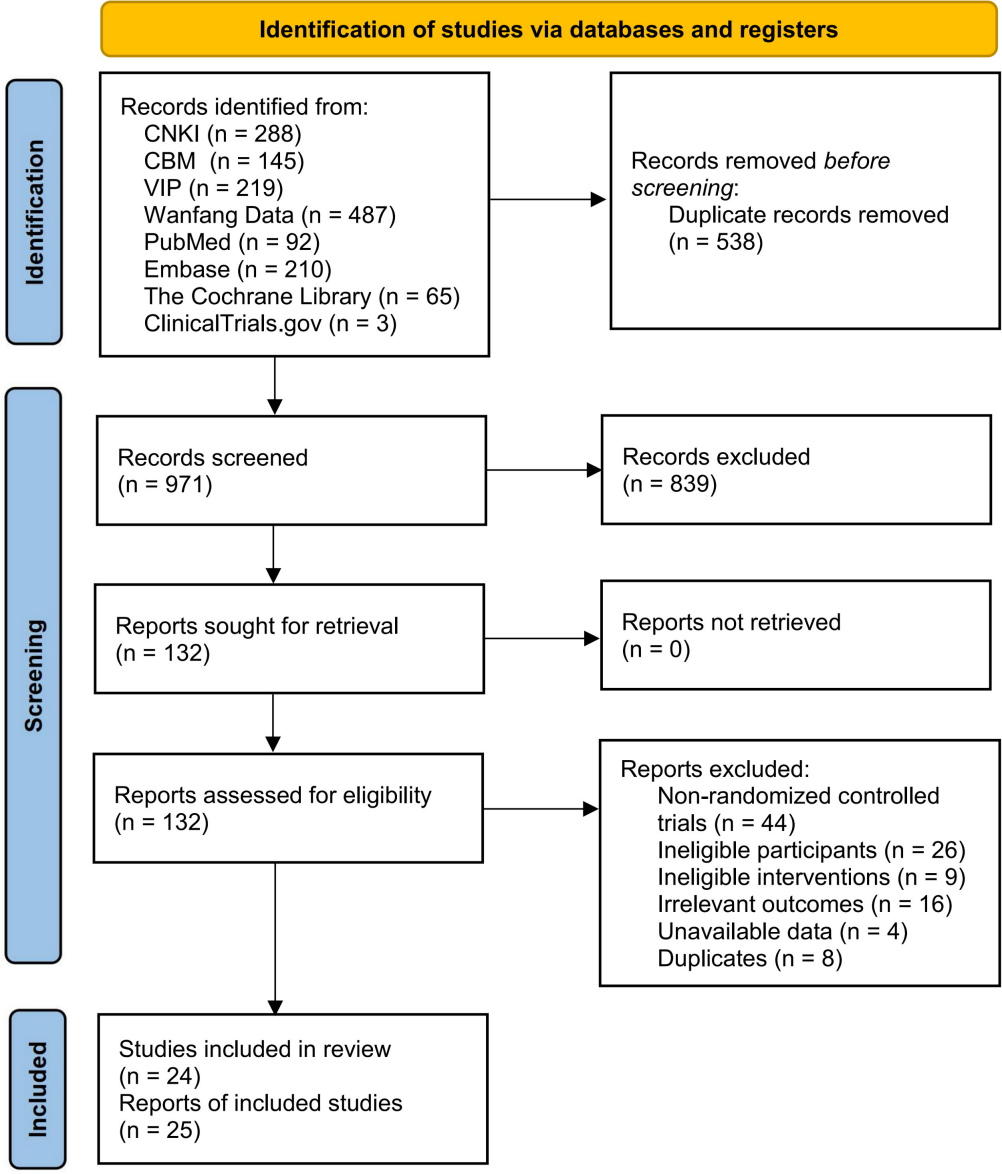
PRISMA flow diagram of the selection process.

A total of 1,506 records were searched from databases and three records from websites. After removing 538 duplicated records, we further excluded irrelevant 839 records. Finally, we included 25 reports (Qin et al., 2000; Zhou, 2003, 2004; Chan et al., 2004; Zhou et al., 2005; Gao, 2006; Woo et al., 2007; Mao, 2009; Liu, 2010; Shen et al., 2010, 2012; Song et al., 2010, 2018; Zhu, 2011; Wayne et al., 2012; Kuo et al., 2014; Yu et al., 2014; Lu and Song, 2015; Wang et al., 2015; Xue, 2015; Ye et al., 2016; Xu, 2017; Cheng and Ba, 2020; Zhang, 2020; Zou, 2020), involving 24 studies through full-text reading (Figure 1). The list of excluded records with reasons is provided in Appendix 3. Shen et al. (2010, 2012) pertained to the same study. Among included studies, two studies was undertaken in America (Shen et al., 2010, 2012; Wayne et al., 2012), one from South Korea (Song et al., 2010), and the others were in China. Eight reports were published in English journals (Chan et al., 2004; Woo et al., 2007; Shen et al., 2010, 2012; Song et al., 2010; Wayne et al., 2012; Wang et al., 2015; Cheng and Ba, 2020), 9 were in Chinese journals (Zhou, 2003, 2004; Zhou et al., 2005; Mao, 2009; Yu et al., 2014; Lu and Song, 2015; Ye et al., 2016; Xu, 2017; Song et al., 2018), six were master's theses (Gao, 2006; Liu, 2010; Zhu, 2011; Xue, 2015; Zhang, 2020; Zou, 2020), and two were conference abstracts (Qin et al., 2000; Kuo et al., 2014). The duration of Tai Chi practice ranged from 2 to 24 months, and sample size varied from 16 to 344. Sixteen studies evaluated the BMD by dual-energy x-ray absorptiometry (Qin et al., 2000; Zhou, 2003, 2004; Chan et al., 2004; Zhou et al., 2005; Woo et al., 2007; Mao, 2009; Song et al., 2010, 2018; Wayne et al., 2012; Kuo et al., 2014; Yu et al., 2014; Wang et al., 2015; Ye et al., 2016; Xu, 2017; Cheng and Ba, 2020), six used calcaneus quantitative ultrasound (Gao, 2006; Liu, 2010; Zhu, 2011; Lu and Song, 2015; Zhang, 2020; Zou, 2020), and four observed the change of bone turnover markers (Liu, 2010; Shen et al., 2010, 2012; Wayne et al., 2012; Xue, 2015). Table 1 provides the characteristics of included studies.

**Table 1:** Characteristics of included studies.

**Study**	**Site**	**Total sample size** **(Randomized/** **analyzed)**	**Sample size of each** **group (Randomized/** **analyzed)**	**Age**	**Intervention**		**Comparator**		**Duration**	**Outcome**
					**Type**	**Frequency**	**Type**	**Frequency**		
Chan et al. (2004)	China (Hong Kong)	132/103	E: 67/54 C: 65/49	E: 54.4 ± 3.3 C: 53.6 ± 3.2	Yang-style Tai Chi (Quan)	50 min/time, 5 times/week	Non-intervention	NA	12 months	BMD (Lumbar spine) BMD (Femoral neck) BMD (Trochanter)
Cheng and Ba (2020)	China	44/34	E: 22/17 C: 22/17	E: 61.3 ± 2.4 C: 61.9 ± 2.5	24-style Tai Chi (Quan)	40 min/time, 5 times/week	Non-intervention	NA	12 months	BMD (Lumbar spine) BMD (Femoral neck) BMD (Ward's triangle) BMD (Trochanter)
Gao (2006)	China	48/48	E: 16/16 C1: 16/16 C2: 16/16	E: 63.8 ± 3.5 C1: 64.2 ± 3.9 C2: 64.1 ± 3.7	36-style Tai Chi (Fan)	60 min/time, 7 times/week	C1: Non-intervention C2: Dancing	C1: NA C2: 60 min/time, 7 times/week	10 weeks	BMD of calcaneus BQI BUA SOS
Kuo et al. (2014)	China (Tai Wan)	75/61	E: NI/21 C1: NI/20 C2: NI/20	NI	Tai Chi (Quan) + calcium and vitamin D supplements	NI	C1: Calcium and vitamin D supplements C2: Aerobic training + resistance training + stretching + calcium and vitamin D supplements	C1: NI C2: 60 min/time, 3 times/week	3 months	BMD (Lumbar spine) BMD (Femoral neck)
Liu (2010)	China	60/45	E: 20/16 C1: 20/14 C2: 20/15	E: 63.7 ± 3.1 C1: 62.4 ± 2.3 C2: 63.2 ± 2.8	24-style Tai Chi (Quan)	55-60 min/time, ≥ 5 times/week	C1: Non-intervention C2: Brisk walking	C1: NA C2: 100-140 steps/min, 55-60 min/time	4 months	BQI BUA SOS ALP
Lu and Song (2015)	China	70/61	E: 35/31 C: 35/30	E: 62.1 ± 5.5 C: 62.9 ± 5.3	Chen-style Tai Chi (Quan)	40 min/time, 6 times/week	Brisk walking	40 min/time, 6 times/week	12 months	BQI
Mao (2009)	China	80/80	E1: 20/20 E2: 20/20 C1: 20/20 C2: 20/20	56.8 ± 2.9	E1: Tai Chi (Quan) E2: Tai Chi (Quan) + calcium-vitamin D chewable tablet	45-50 min/time	C1: Non-intervention C2: Calcium-vitamin D chewable tablet	C1: NA C2: 2 tablets/time, 1 time/day	5 months	BMD (Lumbar spine)
Qin et al. (2000)	China (Hong Kong)	164/99	E: 93/45 C: 71/54	NI	85-style Tai Chi (Quan)	45 min/time, 5 times/week	Non-intervention	NA	12 months	BMD (Lumbar spine) BMD (Femoral neck) BMD (Ward's triangle) BMD (Trochanter)
Shen et al. (2012)	America	171/150	E1: 42/37 E2: 38/37 C1: 44/37 C2: 47/39	E1: 58.3 ± 7.7 E2: 57.6 ± 6.7 C1: 57.6 ± 7.5 C2: 56.5 ± 5.5	E1: 24-style Tai Chi (Quan) + placebo + calcium and vitamin D supplements E2: 24-style Tai Chi (Quan)+ green tea polyphenols + calcium and vitamin D supplements	60 min/time, 3 times/week	C1: Placebo + calcium and vitamin D supplements C2: Green tea polyphenols + calcium and vitamin D supplements	C1: 1 capsule (medicinal starch)/time, 2 times/day + 500 mg elemental calcium and 200 IU vitamin D daily C2: 1 capsule (green tea polyphenols)/time, 2 times/day + 500 mg elemental calcium and 200 IU vitamin D daily	6 months	BAP TRAP
Shen et al. (2010)	America	171/171	E1: 42/42 E2: 38/38 C1: 44/44 C2: 47/47	E1: 58.3 ± 7.7 E2: 57.6 ± 6.7 C1: 57.6 ± 7.5 C2: 56.5 ± 5.5	E1: 24-style Tai Chi (Quan) + placebo + calcium and vitamin D supplements E2: 24-style Tai Chi (Quan)+ green tea polyphenols + calcium and vitamin D supplements	60 min/time, 3 times/week	C1: Placebo + calcium and vitamin D supplements C2: Green tea polyphenols + calcium and vitamin D supplements	C1: 1 capsule (medicinal starch)/time, 2 times/day + 500 mg elemental calcium and 200 IU vitamin D daily C2: 1 capsule (green tea polyphenols)/time, 2 times/day + 500 mg elemental calcium and 200 IU vitamin D daily	6 months	ALP
Song et al. (2018)	China	106/88	E: 35/28 C1: 36/31 C2: 35/29	E: 64.3 ± 3.2 C1: 64.7 ± 4.1 C2: 64.8 ± 2.9	24-style Tai Chi (Quan)	70 min/time, 5 times/week	C1: Non-intervention C2: Brisk walking	C1: NA C2: ≥ 90 steps/min, 70 min/time, 5 times/week	12 months	BMD (Lumbar spine) BMD (Femoral neck) BMD (Ward's triangle) BMD (Trochanter)
Song et al. (2010)	South Korea	82/65	E: 41/30 C: 41/35	E: 63.0 ± 7.3 C: 61.2 ± 8.0	31-style Tai Chi (Quan)	Stage 1 (1-3 weeks): 2 times/week Stage 2 (4-24 weeks): 55-65 min/time, 1 time/week at learning centre + ≥ 20 min at home daily	Self-help education program	2 hours/month	6 months	BMD (Femoral neck) BMD (Ward's triangle) BMD (Trochanter)
Wang et al. (2015)	China	79/69	E: 40/34 C: 39/35	E: 58.5 ± 3.4 C: 58.5 ± 3.4	Yang style Tai Chi (Quan)	60 min/time, 4 times/week	Non-intervention	NA	12 months	BMD (Lumbar spine) BMD (Femoral neck) BMD (Ward's triangle)
Wayne et al. (2012)	America	86/86	E: 43/43 C: 43/43	E: 58.8 ± 5.6 C: 60.4 ± 5.3	Wu/Yang style Tai Chi (Quan) + standard care	30-60 min/time, 4 times/week	Standard care (calcium and vitamin D supplements + regular exercise)	NI	9 months	BMD (Femoral neck) BMD (Total hip) BMD (Lumbar spine) CTX OSC
Woo et al. (2007)	China (Hong Kong)	90/88	E: 30/28 C1: 30/30 C2: 30/30	E: 69.7 ± 2.8 C1: 69.3 ± 3.0 C2: 69.6 ± 3.2	24-style Tai Chi (Quan)	3 times/week	C1: Non-intervention C2: Resistance exercise	C1: NA C2: 3 times/week	12 months	BMD (Total hip) BMD (Total spine)
Xu (2017)	China	86/86	E: 43/43 C: 43/43	E: 56.2 ± 5.6 C: 57.1 ± 6.0	24-style Tai Chi (Quan)	≥ 40 min/time, ≥ 6 times/week	Non-intervention	NA	12 months	BMD (Lumbar spine) BMD (Femoral neck) BMD (Ward's triangle) BMD (Trochanter)
Xue (2015)	China	344/283	E: 171/136 C: 173/147	E: 62.1 ± 7.0 C: 64.0 ± 7.3	Tai Chi (Quan) + education	30 min/time, 3-5 times/week	Education	NI	24 months	PINP CTX
Ye et al. (2016)	China	50/39	E: 25/17 C: 25/22	E: 55.4 ± 6.1 C: 57.0 ± 8.5	Tai Chi (Quan)	≥ 60 min/time, 3 times/week	Non-intervention	NA	6 months	BMD (Lumbar spine) BMD (Femoral neck) BMD (Ward's triangle)
Yu et al. (2014)	China	77/61	E: 38/30 C: 39/31	E: 59.2 ± 3.6 C: 58.5 ± 3.5	8/24/42-style Tai Chi (Quan)	60 min/time, 4 times/week	Non-intervention	NA	12 months	BMD (Lumbar spine) BMD (Trochanter) BMD (Ward's triangle)
Zhang (2020)	China	40/37	E: 20/17 C: 20/20	E: 56.3 ± 5.2 C: 55.1 ± 6.7	32-style Tai Chi (Sword)	60 min/time, 5 times/week	Non-intervention	NA	3 months	BQI BMD of calcaneus
Zhou (2003)	China	36/34	E: 12/12 C1: 12/10 C2: 12/12	E: 57.1 ± 2.7 C1: 56.0 ± 2.8 C2: 56.8 ± 2.6	Tai Chi (Push hands)	45-60 min/time, 5-7 times/week	C1: Non-intervention C2: Walking + running	C1: NA C2: 45-60 min/time, 5-7 times/week	10 months	BMD (Lumbar spine)
Zhou (2004)	China	36/36	E: 12/12 C1: 12/12 C2: 12/12	55.9 ± 2.8	24/42-style Tai Chi (Quan)	45-60 min/time, 5-7 times/week	C1: Non-intervention C2: Rope skipping	C1: NA C2: 45 min/time, 5-7 times/week	10 months	BMD (Lumbar spine)
Zhou et al. (2005)	China	64/64	E1: 16/16 E2: 16/16 C1: 16/16 C2: 16/16	57.2 ± 3.4	E1: Tai Chi (Push hands) E2: Tai Chi (Push hands) + calcium-vitamin D chewable tablet	45-60 min/time, 5-7 times/week	C1: Non-intervention C2: Calcium-vitamin D chewable tablet	C1: NA C2: 1 tablet/time, 2 times/day	6 months	BMD (Lumbar spine)
Zhu (2011)	China	16/16	E: 8/8 C1: 8/8 C2: 8/8	E: 50.5 ± 3.1 C1: 50.8 ± 2.8 C2: 51.9 ± 3.4	Tai Chi (Quan)	25 min/time, 3 times/week	C1: Non-intervention C2: Rope skipping	C1: NA C2: 0.5 min/set, 25 sets/time, 3 times/week	6 months	BUA SOS BQI
Zou (2020)	China	28/28	E: 10/10 C1: 8/8 C2: 10/10	E: 65.1 ± 4.4 C1: 65.6 ± 2.7 C2: 65.3 ± 5.2	Tai Chi (Quan)	60 min/time, 3 times/week	C1: Non-intervention C2: Resistance training	C1: NA C2: 60 min/time, 3 times/week	2 months	BMD of calcaneus

E, experimental group; C, control group; min, minutes; BMD, bone mineral density; BQI, bone quality index; BUA, broadband ultrasonic attenuation; SOS, speed of sound; PINP, Procollagen type I N-terminal propeptide; CTX, C-terminal telopeptide of type I collagen; ALP, alkaline phosphatase; TRAP, tartrate-resistant acid phosphatase; BAP, bone-specific alkaline phosphatase; OSC, osteocalcin; MD, mean difference; NA, not applicable; NI, no information.

### Risk of bias

**Figure 2:**
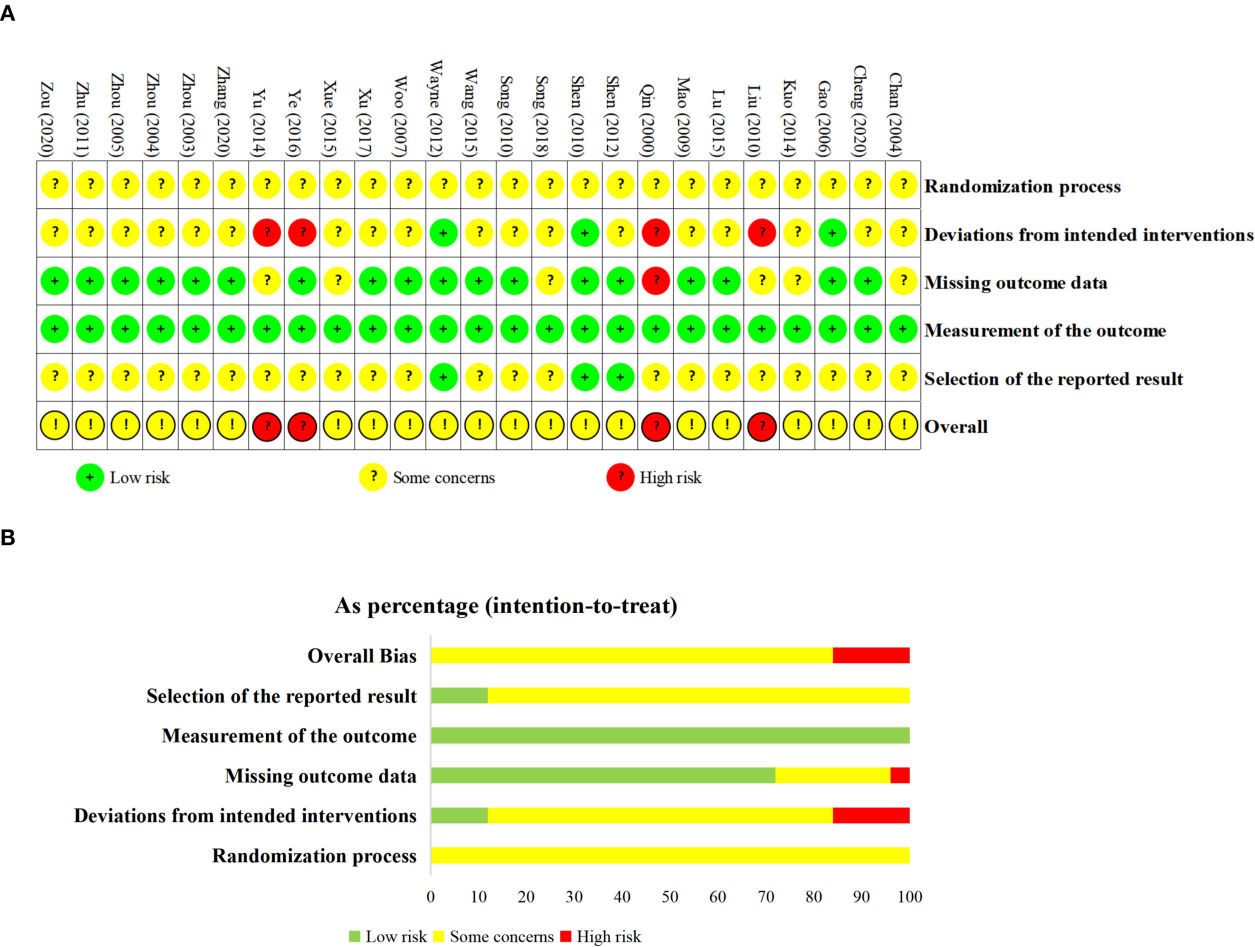
Results of risk of bias assessment. **(A)** the domain and overall judgments study-by-study; **(B)** proportions of studies at low risk, some concerns or high risk of bias for each domain.

Seven studies (Woo et al., 2007; Song et al., 2010, 2018; Wayne et al., 2012; Lu and Song, 2015; Ye et al., 2016; Xu, 2017) specified the methods of randomization, and all studies did not provide information about allocation concealment. Seventeen studies (Qin et al., 2000; Zhou, 2003; Chan et al., 2004; Woo et al., 2007; Liu, 2010; Shen et al., 2010, 2012; Song et al., 2010, 2018; Wayne et al., 2012; Kuo et al., 2014; Yu et al., 2014; Lu and Song, 2015; Wang et al., 2015; Xue, 2015; Ye et al., 2016; Cheng and Ba, 2020; Zhang, 2020) reported the number of drop-outs or lost to follow-up. Two (Shen et al., 2010; Wayne et al., 2012) studies performed the intent-to-treat analysis and the remaining studies used per-protocol analysis. The primary and secondary outcomes were objective indicators. Two studies (Shen et al., 2010, 2012; Wayne et al., 2012) provided the registration numbers. In summary, four studies (Qin et al., 2000; Liu, 2010; Yu et al., 2014; Ye et al., 2016) were considered as high overall risk of bias, and the rest of the studies were rated as some concerns. The results of the ROB assessment are shown in Figure 2.

## Meta-analysis

### BMD

#### Tai Chi vs. non-intervention

**Figure 3:**
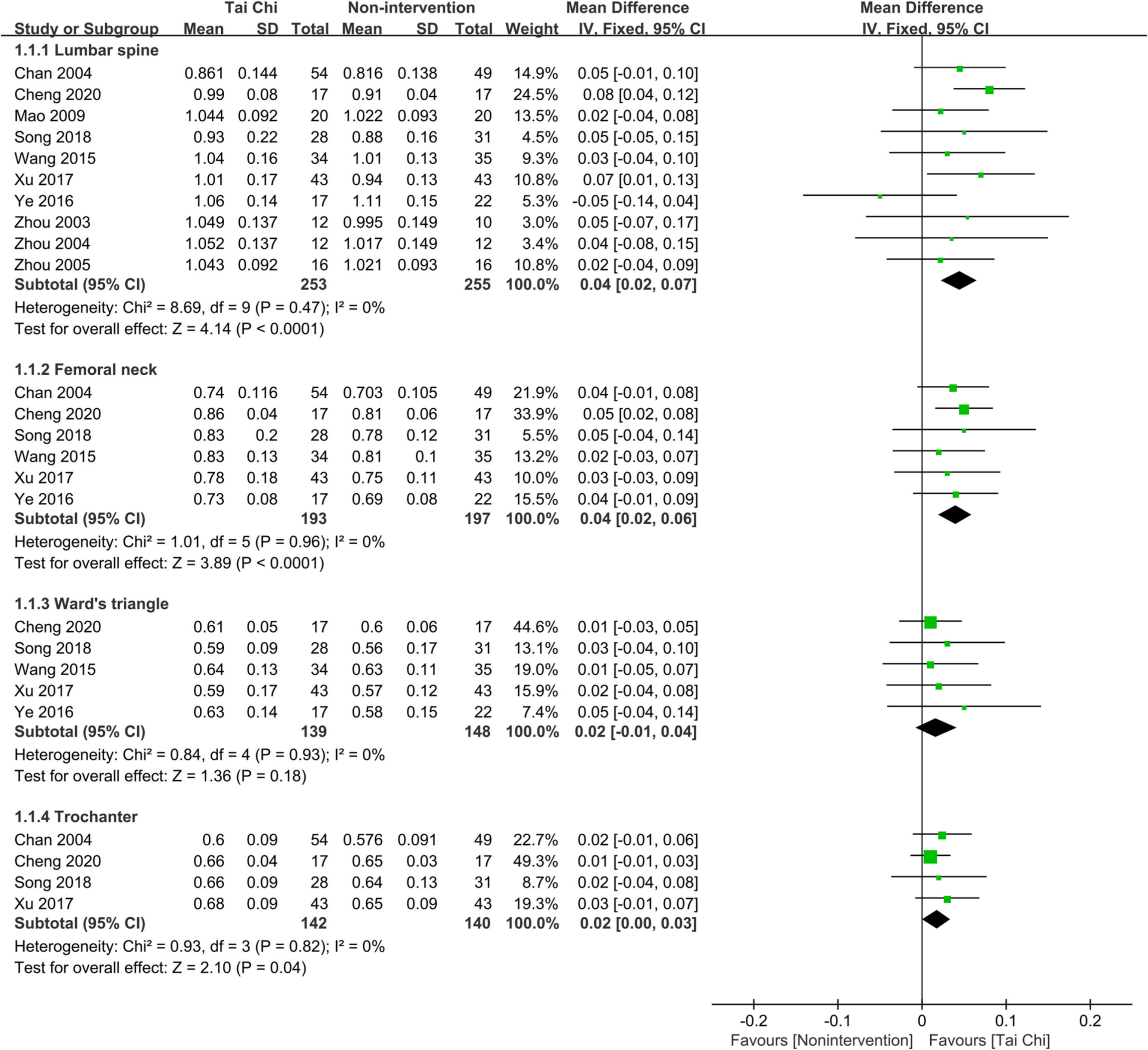
Forest plot of meta-analysis results for Tai Chi vs. non-intervention.

Compared with non-intervention group, participants in Tai Chi group had higher BMD of lumbar spine (MD = 0.04, 95% CI 0.02 to 0.07, *P* < 0.0001, *I*^2^ = 0%), femoral neck (MD = 0.04, 95% CI 0.02 to 0.06, *P* < 0.0001, *I*^2^ = 0%), and trochanter (MD = 0.02, 95% CI 0.00 to 0.03, *P* = 0.04, *I*^2^ = 0%). Notwithstanding, there was no difference between Tai Chi and non-intervention group in the BMD of Ward's triangle (MD = 0.02, 95% CI −0.01 to 0.04, *P* = 0.18, *I*^2^ = 0%) (Figure 3).

Results of BMD in the lumbar spine, femoral neck, and Ward's triangle were stable after excluding studies one by one. But the pooled result of trochanter altered to insignificant after excluding Chan et al. (2004) (MD = 0.02, 95% CI −0.00 to 0.04, *P* = 0.10, *I*^2^ = 0%) or Xu (2017) (MD = 0.02, 95% CI −0.00 to 0.03, *P* = 0.11, *I*^2^ = 0%). The plots of sensitivity analysis results are shown in Figure S1 in Appendix 4.

Three studies (Qin et al., 2000; Woo et al., 2007; Yu et al., 2014) evaluated the percentage change of BMD. No differences between Tai Chi and non-intervention group were found in the percentage change of BMD in lumbar spine (MD = 0.83, 95% CI −0.12 to 1.77, *P* = 0.09, *I*^2^ = 28%), Ward's triangle (MD = 1.81, 95% CI −0.28 to 3.90, *P* = 0.09, *I*^2^ = 31%) and trochanter (MD = −0.07, 95% CI −1.35 to 1.22, *P* = 0.92, *I*^2^ = 0%) (Figure S2 in Appendix 4). Two studies reported that the percentage change of BMD of femoral neck (Qin et al., 2000) and total spine (Woo et al., 2007) in the Tai Chi group did not differ from the non-intervention group. However, Woo et al. (2007) found that Tai Chi could attenuate greater BMD loss of total hip than non-intervention.

One study (Song et al., 2010) showed that the improvement of BMD T score of femoral neck, Ward's triangle, and trochanter was significantly higher in the Tai Chi group than that in the education program group.

#### Tai Chi vs. other exercises

**Figure 4:**
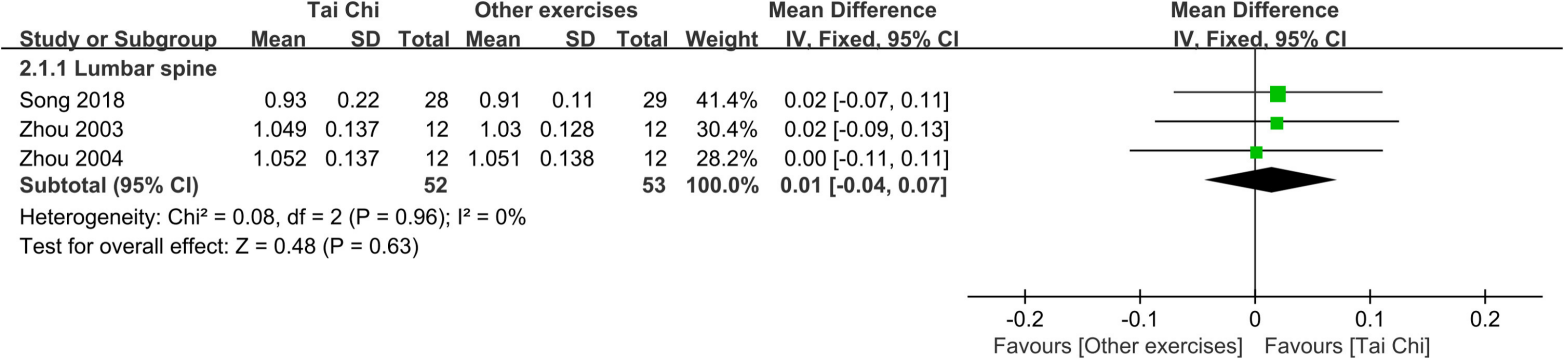
Forest plot of meta-analysis results for Tai Chi vs. other exercises.

There were no differences between Tai Chi and other exercises in increasing BMD of lumbar spine (MD = 0.01, 95% CI −0.04 to 0.07, *P* = 0.63, *I*^2^ = 0%) (Figure 4). The result did not change during sensitivity analysis (Figure S3 in Appendix 4).

Song et al. (2018) observed Tai Chi did not differ from brisk walking in improving the BMD of femoral neck, Ward's triangle and trochanter. Woo et al. (2007) found no difference existed in the percentage change of BMD of total hip between Tai Chi and resistance exercise group, and resistance exercise increased more BMD of total spine than Tai Chi. Kuo et al. (2014) reported that Tai Chi plus calcium and vitamin D supplements was not superior to circuit training program (aerobic training, resistance training, and stretching) plus calcium and vitamin D supplements in increasing BMD of femoral neck and lumbar spine.

#### Tai Chi plus nutraceutical vs. nutraceutical

**Figure 5:**
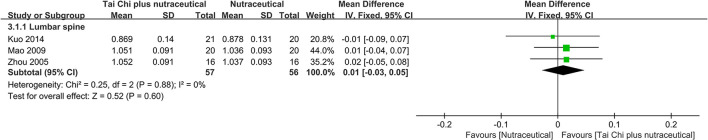
Forest plot of meta-analysis results for Tai Chi plus nutraceutical vs. nutraceutical.

No significant difference between Tai Chi plus nutraceutical and nutraceutical was found in BMD of lumbar spine (MD = 0.01, 95% CI −0.03 to 0.05, *P* = 0.60, *I*^2^ = 0%) (Figure 5). The results remained unchanged according to the sensitivity analysis (Figure S4 in Appendix 4).

Kuo et al. (2014) observed that the BMD of femoral neck did not differ significantly between Tai Chi plus nutraceutical group and nutraceutical group. Wayne et al. (2010) reported there was no difference in BMD of lumbar spine, femoral neck, and total hip between Tai Chi plus standard care and standard care group.

### Calcaneus quantitative ultrasound

#### Tai Chi vs. non-intervention

Compared with the non-intervention group, the Tai Chi group had a significantly greater speed of sound, while had no difference in BMD of calcaneus, broadband ultrasonic attenuation, and bone quality index (Table 2). Based on sensitivity analysis, the results of speed of sound (MD = 17.09, 95% CI −1.09 to 35.28, P = 0.07, *I*^2^ = 0%) became non-significant after excluding Gao (2006) (Figure S5 in Appendix 4).

**Table 2:** The meta-analysis results for outcomes of calcaneus quantitative ultrasound.

**Outcome**	**Comparison**	**Number of studies**	**Analyzed subjects**	**Overall effect**		**Heterogeneity**	
				**MD (95% CI)**	***P***	***I^2^***	***P***
BMD of calcaneus	Tai Chi vs. non-intervention	3	87	0.22 (−0.22 to 0.66)	0.32	76%	0.02
	Tai Chi vs. other exercises	2	52	0.12 (−0.14 to 0.38)	0.35	0%	0.72
BQI	Tai Chi vs. non-intervention	4	115	4.19 (−3.65 to 12.03)	0.29	80%	0.002
	Tai Chi vs. other exercises	4	140	3.12 (−1.23 to 7.46)	0.16	0%	0.53
BUA	Tai Chi vs. non-intervention	3	78	6.79 (0.01 to 13.56)	0.05	57%	0.10
	Tai Chi vs. other exercises	3	79	1.30 (−5.01 to 7.62)	0.69	51%	0.13
SOS	Tai Chi vs. non-intervention	3	78	20.83 (10.44 to 31.22)	<0.0001	0%	0.65
	Tai Chi vs. other exercises	3	79	5.46 (−19.90 to 30.81)	0.67	74%	0.02

BMD, bone mineral density; BQI, bone quality index; BUA, broadband ultrasonic attenuation; SOS, speed of sound; MD, mean difference.

#### Tai Chi vs. other exercises

There were no significant differences in the BMD of calcaneus, speed of sound, broadband ultrasonic attenuation, and bone quality index between Tai Chi and other exercises (Table 2). And the above results did not alter after excluding studies one by one. The plots of sensitivity analysis are shown in Figure S6 in Appendix 4.

### Bone turnover markers

Xue (2015) found that the Tai Chi plus education group had a higher level of serum PINP than the education group, but there was no difference in the level of serum CTX. Liu (2010) observed no difference of the comparisons of Tai Chi vs. non-intervention and Tai Chi vs. brisk walking in the level of serum ALP. Two articles reported Tai Chi plus nutraceutical group was not superior to the nutraceutical group in the level of serum ALP (Shen et al., 2010), BAP (Shen et al., 2012), TRAP (Shen et al., 2012). There were no differences between Tai Chi plus standard care and standard care in level of serum OSC (Wayne et al., 2012) and CTX (Wayne et al., 2012).

### Subgroup analysis

Compared with non-intervention, practicing Tai Chi for more than 6 months showed greater BMD of the lumbar spine, femoral neck, and trochanter, while practicing it for less than or equal to 6 months was not superior to non-intervention in increasing BMD of the lumbar spine, femoral neck, and Ward's triangle (Table 3). The forest plots of subgroup analyses are shown in Figure S7 in Appendix 4.

**Table 3:** Subgroup analysis according to the duration of Tai Chi compared with non-intervention.

**Outcome**	**Duration of Tai Chi**	**Number of studies**	**Analyzed subjects**	**Overall effect**		**Heterogeneity**	
				**MD (95% CI)**	***P***	***I^2^***	***P***
BMD (Lumbar spine)	≤ 6 months	3	111	0.01 (−0.03 to 0.05)	0.65	0%	0.38
	> 6 months	7	397	0.06 (0.03 to 0.08)	<0.00001	0%	0.90
BMD (Femoral neck)	≤ 6 months	1	39	0.04 (−0.01 to 0.09)	0.12	-	-
	> 6 months	5	351	0.04 (0.02 to 0.06)	0.0004	0%	0.91
BMD (Ward's triangle)	≤ 6 months	1	39	0.05 (−0.04 to 0.14)	0.28	-	-
	> 6 months	4	248	0.01 (−0.01 to 0.04)	0.27	0%	0.96
BMD (Trochanter)	≤ 6 months	-	-	-	-	-	-
	> 6 months	4	282	0.02 (0.00 to 0.03)	0.04	0%	0.82

BMD, bone mineral density; MD, mean difference.

### Adverse events

Four studies (Shen et al., 2010, 2012; Wayne et al., 2012; Wang et al., 2015; Xue, 2015) stated no adverse events were attributed to Tai Chi practice. Chan et al. (2004) reported that one proximal fibular fracture occurred in the Tai Chi group due to a fall. Woo et al. (2007) observed no significant difference in the number of falls between Tai Chi, resistance exercise, and non-intervention groups during the study period. The remaining studies did not provide any information about adverse events.

### TSA

According to TSA of Tai Chi vs. non-intervention, the included sample size reached the required information size in BMD of the lumbar spine (508 vs. 197) and femoral neck (390 vs. 197). Therefore, there was sufficient evidence favoring the effect of Tai Chi on BMD of the lumbar spine and femoral neck. However, the included sample size of BMD of Ward's triangle (287 vs. 785) and trochanter (282 vs. 785) did not achieve the required information size, and their cumulative Z curves did not cross the trial sequential monitoring boundaries or futility boundaries. Thus more studies are needed to verify the effect of Tai Chi on Ward's triangle and trochanter (Appendix 5).

### Publication bias

**Figure 6:**
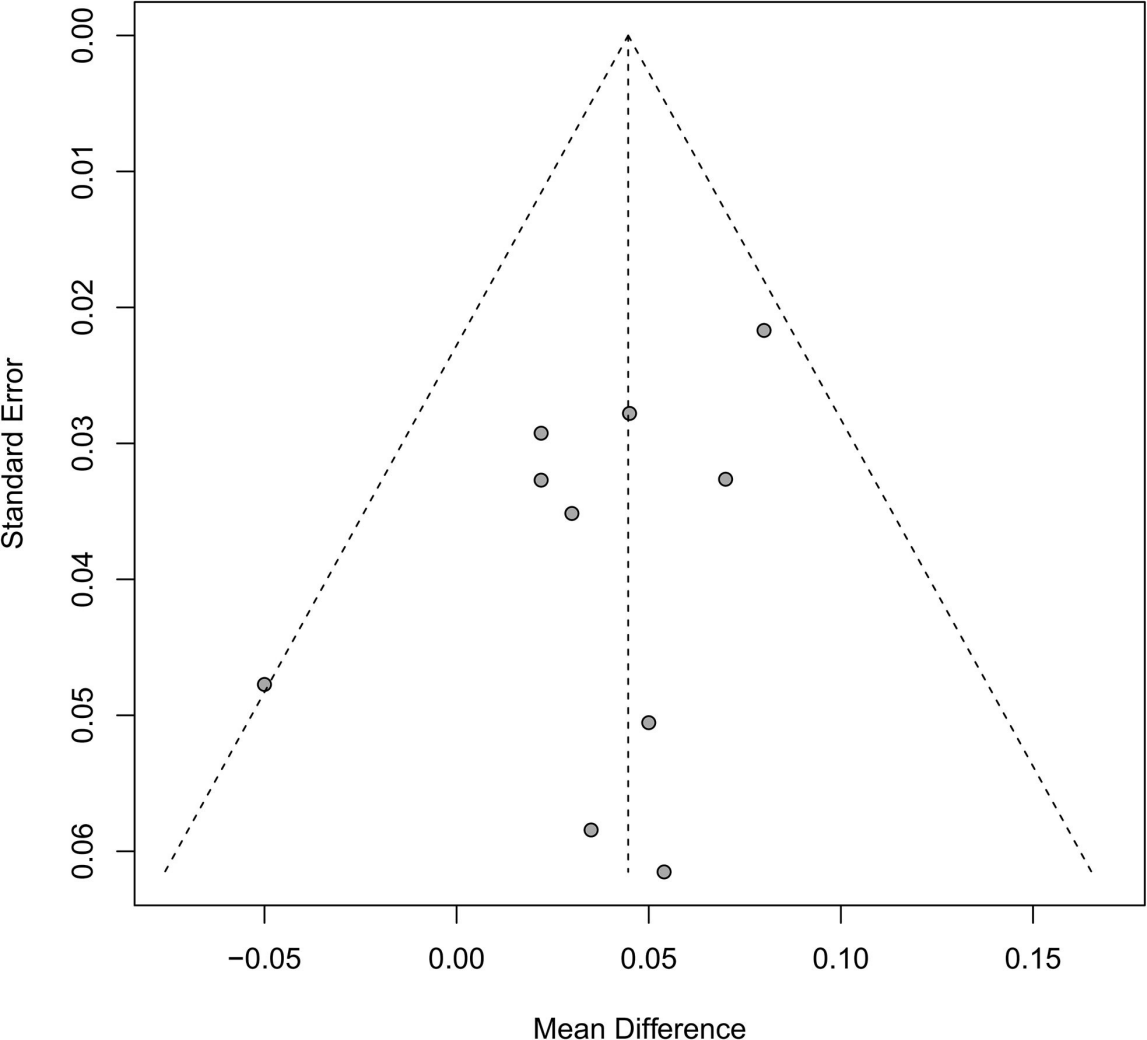
The funnel plot of BMD of lumbar spine of Tai Chi vs. non-intervention.

Ten studies reported the BMD of lumbar spine of Tai Chi vs. non-intervention, thus we evaluated the publication bias. The funnel plot (Figure 6) and *Egger's* test (*P* = 0.17) suggested no evidence of publication bias existed.

### Certainty of evidence

The results of certainty of evidence are shown in Appendix 6. The certainty of evidence for three outcomes (BMD of the lumbar spine and femoral neck of Tai Chi vs. non-intervention, and BMD of the lumbar spine of Tai Chi practicing for over 6 months vs. non-intervention) was graded as low. The evidence of the remaining outcomes was rated as very low certainty. The reasons for downgrading were mainly attributed to the risk of bias of included studies, imprecision and publication bias.

## Discussion

### Summary of findings

In this meta-analysis and TSA, we included 24 studies that investigated the effect and safety of Tai Chi on bone health in postmenopausal women. We found that Tai Chi training was superior to non-intervention in improving BMD of the lumbar spine and femoral neck, and the above evidence was reliable according to TSA. Tai Chi might improve the BMD of trochanter better than non-intervention, but sensitivity analysis and TSA indicated the result needed further verification. There were no significant differences in BMD of the lumbar spine, femoral neck, trochanter, and Ward's triangle when comparing Tai Chi with other exercises. Tai Chi plus nutraceuticals also did not differ from nutraceuticals in improving BMD of the lumbar spine and femoral neck. Insufficient data was obtained to support the effect of Tai Chi on bone turnover markers and calcaneus quantitative ultrasound. Subgroup analysis demonstrated that practicing Tai Chi for over 6 months improved more BMD of the lumbar spine, femoral neck, and trochanter than non-intervention. The certainty of evidence was low for three outcomes, including the BMD of the lumbar spine and femoral neck of Tai Chi vs. non-intervention and the BMD of the lumbar spine of Tai Chi practicing for over 6 months vs. non-intervention. The certainty of evidence of the rest outcomes was very low. Few Tai Chi-related adverse events occurred.

### Comparison with other SRs

Previous relevant SRs hold contradictory conclusions. Liu and Wang (2017) included 350 participants from six studies published up to 2016 and concluded that Tai Chi was not effective to attenuate BMD loss of the lumbar spine and femoral neck in postmenopausal women. Sun et al. (2016) pooled data from six studies before May 2015 and reported that Tai Chi had a significant effect on BMD of the lumbar spine when compared with no treatment, which was consistent with our results. While, Sun et al. (2016) included perimenopausal and postmenopausal women. Furthermore, Sun et al. (2016) double-counted the participants in the control group from a 3-arm study (Zhou, 2004), which might introduce a unit-of-analysis error (Rücker et al., 2017). Compared to Sun et al. (2016) and Liu and Wang (2017), we updated more RCTs, introduced more outcomes, validated results with TSA, and confirmed the effect of Tai Chi on BMD of lumbar spine and femoral neck in postmenopausal women.

### Implications for clinical practice and future studies

During Tai Chi exercise, the practitioners hold a half-squat posture and switch between double-stance and single-stance weight-bearing, along with pivoting and twisting the trunk. Researchers found Tai Chi movements could produce vertical weight-bearing force and activate the lumbar erector spine muscle and lower extremity muscle (Chan et al., 2003; Wu and Hitt, 2005). Compared with a normal gait, the Tai Chi gait had a greater peak shear force and larger frontal-plane joint moment in the hip (Wu and Millon, 2008; Yang and Liu, 2020). Previous meta-analysis (Yang et al., 2021) demonstrated that Tai Chi could improve the thoracolumbar spine flexibility and enhance lower limb muscle strength. In our study, we found Tai Chi increased more BMD of the lumbar spine, femoral neck, and trochanter. However, sensitivity analysis suggested the effect of Tai Chi for the BMD in trochanter was unstable. Additionally, Tai Chi was not superior to non-intervention in improving the BMD of Ward's triangle. We speculated that the reason might be related to the small sample size.

In our study, Tai Chi-induced BMD gain in the lumbar spine and femoral neck was 0.04 g/cm^2^ when compared with non-intervention. Since the minimum clinically important difference (MCID) for BMD was not reported, we failed to determine the clinical significance. While Chen et al. (2006) found that an increase in lumbar spine BMD of 0.09 g/cm^2^ reduced the risk of vertebral fracture in postmenopausal women with osteoporosis by 30–41%, Jacques et al. (2012) reported that among postmenopausal women with osteoporosis, patients with 3-year increase in BMD of 0–0.032 and 0.032 g/cm^2^ were 0.48 and 0.27 times more likely to suffer from vertebral fracture than those with change of BMD <0 g/cm^2^, respectively. Future studies are needed to establish an MCID for BMD of different sites in postmenopausal women.

During bone remodeling, bone resorption lasts 4–6 weeks and subsequently bone formation maintains 4–5 months (Eastell and Szulc, 2017). Thus, in the previous SRs exploring the effect of exercise on BMD (Zhao et al., 2014, 2015; Kemmler et al., 2020; Mohammad Rahimi et al., 2020), they preferred to include trials in which exercise lasted for at least 6 months. Our results also found that practicing Tai Chi for less than or equal to 6 months had no effect on BMD while practicing Tai Chi over 6 months could improve more BMD than non-intervention. However, it must be acknowledged that our subgroup analysis of Tai Chi practicing for less than or equal to 6 months vs. non-intervention included few RCTs, which might decrease the statistical power.

Among included studies, the control exercises involved aerobic exercise combined with resistance training (Kuo et al., 2014), rope skipping (Zhou, 2004), running combined with walking (Zhou, 2003), and brisk walking (Song et al., 2018). These exercises were reported to improve BMD in premenopausal women (Pellikaan et al., 2018; Kemmler et al., 2020; Lan and Feng, 2022). Our results showed that Tai Chi was an effective exercise to increase BMD of lumbar spine and femoral neck, but no better than other exercises. Results of a network meta-analysis (Zhang et al., 2021) showed that mind-body exercise was the optimal exercise type to improve the BMD of the lumbar spine and femoral neck, while aerobic exercise and resistance exercise had a better effect on BMD of the total hip than mind-body exercise. It was inferred that different exercise patterns might have advantages in improving the BMD of different sites.

Previous studies demonstrated that nutraceuticals, such as calcium and vitamin D supplements might increase BMD of the lumbar spine and femoral neck among postmenopausal women with osteoporosis (Liu et al., 2020). However, nutraceuticals did not decrease the risk of fractures among community-dwelling older adults (Zhao et al., 2017). Propensity to fall was a significant risk factor for fracture. Tai Chi was reported to improve balance and reduce the incidence of falls in older people by 30% (Li et al., 2018; Zhong et al., 2020). Therefore, it's believed that a combination of Tai Chi and nutraceutical may have potential advantages to prevent falls and fractures.

Calcaneal quantitative ultrasound is an alternative approach to assess bone health, which was suggested for pre-screening and risk evaluation for osteoporosis (Gao et al., 2021; Yen et al., 2021). Notwithstanding, Frost et al. (2001) found that the precision of calcaneal quantitative ultrasound was not good enough to be used for monitoring response to treatment. Moreover, the Chinese Society of Osteoporosis and Bone Mineral Research did not recommend to use quantitative ultrasound for the evaluation of intervention efficacy (Xia et al., 2019). In our study, the results of calcaneal quantitative ultrasound seemed to be erratic, and high heterogeneity existed among studies. Therefore, researchers should combined calcaneal quantitative ultrasound with other more sensitive detection methods (e.g. dual-energy x-ray absorptiometry) to assess the response to intervention.

Due to limited included studies, we failed to confirm the effect of Tai Chi on bone turnover marks quantitatively. Xue (2015) observed that Tai Chi could improve the level of serum PINP. PINP is recommended as the preferred bone formation marker. That might indicate that Tai Chi can promote bone formation to improve the BMD. However, we found Tai Chi had no effect on other bone turnover markers. A recent SR (Kistler-Fischbacher et al., 2021) summarized that there was limited evidence to favor the effect of low-moderate-intensity exercise on bone turnover markers. Future studies can focus on this issue.

## Strengths and limitations

This is the latest meta-analysis of Tai Chi for bone health in postmenopausal women and we performed TSA to explore whether the evidence in our meta-analysis was reliable. However, some limitations should be considered. Firstly, the BMD is a surrogate endpoint for fracture risk, and the MCIDs of BMD of different sites were unclear. Therefore, whether the improvement of BMD that we found would lead to eventual clinical benefits is unknown. Secondly, the optimal protocol of Tai Chi training was not yet been investigated. Thirdly, the majority of included participants were Chinese women, which might limit the general applicability of these results.

## Conclusion

Tai Chi may be an optional and safe exercise for improving BMD loss in postmenopausal women, and practicing Tai Chi for more than 6 months may yield greater benefits. However, more rigorously designed RCTs are required to verify the benefits and to explore the optimal protocol of Tai Chi exercise for bone health.

## Data availability statement

The original contributions presented in the study are included in the article/Supplementary material, further inquiries can be directed to the corresponding authors.

## Author contributions

RJ and JL conceptualized the study and provided methodological support. YL and DZ designed the search strategy. LZ and TaL selected the studies. CJ and RF extracted the data. XL and TiL assessed the risk of bias. XL, CJ, and RF wrote and edited the manuscript. All authors contributed to the article and approved the submitted version.

## Funding

This work was financially funded by the National Natural Science Foundation of China (No: 81873356), the Science and technology project of Sichuan Province (No: 2020YFS0283), Sichuan Province Science and Technology Support Program in Sichuan (No: 2014SZ0154), 2021 Education and Scientific Research Project of National Higher Education of Traditional Chinese Medicine in the 14th Five-Year Plan (No: YB-20-13), and National Natural Science Foundation of China (No: 82104976).

## Conflict of interest

The authors declare that the research was conducted in the absence of any commercial or financial relationships that could be construed as a potential conflict of interest.

## Publisher's note

All claims expressed in this article are solely those of the authors and do not necessarily represent those of their affiliated organizations, or those of the publisher, the editors and the reviewers. Any product that may be evaluated in this article, or claim that may be made by its manufacturer, is not guaranteed or endorsed by the publisher.
