# Supplementary material for: The effect and safety of Tai Chi on bone health in postmenopausal women: A meta-analysis and trial sequential analysis
Source: Front Aging Neurosci. 2022 Sep 13;14:935326. doi: 10.3389/fnagi.2022.935326 (PMC9513206; doi:10.3389/fnagi.2022.935326)
Supplement: Supplementary file 2 [file Table_2.doc]

**Search strategies of all databases**

| **Database** | **Search strategies** |
| --- | --- |
| PubMed | (Bone density[Mesh] OR Osteoporosis[MESH] OR Bone density[TIAB] OR Bone Mineral Density[TIAB] OR bone strength[TIAB] OR bone mass[TIAB] OR bone mineral[TIAB] OR bone tissue[TIAB] OR bone health[TIAB] OR bone turnover markers[TIAB] OR Osteoporosis[TIAB] OR senile osteoporosis[TIAB] OR osteopenia[TIAB]) AND (Tai Ji[MESH] OR Tai Ji[TIAB] OR Tai Chi[TIAB] OR T'ai Chi [TIAB] OR Chi,Tai [TIAB] OR Taijiquan[TIAB] OR Tai Chi quan[TIAB] OR 'Tai Chi Chuan' OR Tai ji Chuan[TIAB]) |
| Embase | #1 'bone density'/exp OR 'osteoporosis'/exp  #2 'bone density':ti,ab,kw OR osteoporosis:ti,ab,kw OR 'bone mineral density':ti,ab,kw OR 'bone strength':ti,ab,kw OR 'bone mass':ti,ab,kw OR 'bone mineral':ti,ab,kw OR 'bone tissue':ti,ab,kw OR 'bone health':ti,ab,kw OR 'bone turnover markers':ti,ab,kw OR 'senile osteoporosis':ti,ab,kw OR osteopenia:ti,ab,kw  #3 'tai chi'/exp  #4 'tai chi chuan':ab,ti,kw OR 'tai chi quan':ab,ti,kw OR 'tai ji':ab,ti,kw OR 'tai ji quan':ab,ti,kw OR 'tai ji chuan':ab,ti,kw OR 'tai chi':ab,ti,kw OR chi,tai:ab,ti,kw  #5 (#1 OR #2) AND (#3 OR #4) |
| The Cochrane library | #1 (bone mineral):ti,ab,kw OR (bone tissue):ti,ab,kw OR (bone health):ti,ab,kw OR (bone turnover markers):ti,ab,kw OR (Osteoporosis):ti,ab,kw (Word variations have been searched)  #2 (senile osteoporosis):ti,ab,kw OR (osteopenia):ti,ab,kw (Word variations have been searched)  #3 MeSH descriptor: [Bone Density] explode all trees  #4 MeSH descriptor: [Osteoporosis] explode all trees  #5 #1 OR #2 OR #3 OR #4  #6 MeSH descriptor: [Tai Ji] explode all trees  #7 (Tai Ji):ti,ab,kw OR (Tai Chi):ti,ab,kw OR (T'ai Chi):ti,ab,kw OR (Taijiquan):ti,ab,kw OR (Tai Chi quan):ti,ab,kw (Word variations have been searched)  #8 (Tai Chi Chuan):ti,ab,kw OR (Tai ji Chuan):ti,ab,kw (Word variations have been searched)  #9 #6 OR #7 OR #8  #10 #5 AND #9 |
| China National Knowledge Infrastructure (CNKI) | TKA= (骨质疏松+骨质疏松症+骨密度+骨代谢+骨健康+骨转换+骨质+骨量+骨组织) AND SU=(太极+太极功法+太极拳） |
| Wanfang Database | 题名或关键词:(太极 or 太极功法 or太极拳) and 主题:(骨质疏松 or 骨质疏松症 or 骨密度 or 骨健康 or 骨转换 or 骨质 or 骨量 or 骨组织 or 骨代谢) |
| Chinese Science and Technology Periodical Database (VIP) | U=(骨质疏松 OR 骨质疏松症 OR 骨密度 OR 骨健康 OR 骨转换 OR 骨质 OR 骨量 OR 骨组织 OR 骨代谢) AND U=(太极 OR 太极功法 OR太极拳) |
| Chinese Biomedical Literature Database (CBM) | #1 "太极拳"[不加权:扩展] OR "太极"[核心字段:智能] OR "太极拳" [核心字段:智能] OR "太极功法"[核心字段:智能]  #2 "骨密度"[不加权:扩展] OR "骨质疏松"[不加权:扩展] OR “骨质疏松"[常用字段:智能OR“骨质疏松症"[常用字段:智能] OR "骨密度"[常用字段:智能] OR "骨健康" [常用字段:智能] OR "骨转换"[常用字段:智能] OR "骨质"[常用字段:智能] OR "骨量"[常用字段:智能] OR "骨组织"[常用字段:智能] OR "骨代谢"[常用字段:智能]  #3 #1 AND #2 |
