# Supplementary material for: The effect and safety of Tai Chi on bone health in postmenopausal women: A meta-analysis and trial sequential analysis
Source: Front Aging Neurosci. 2022 Sep 13;14:935326. doi: 10.3389/fnagi.2022.935326 (PMC9513206; doi:10.3389/fnagi.2022.935326)
Supplement: Supplementary file 3 [file Table_3.DOCX]

**List of excluded studies(n = 107)**

**Non-randomized controlled trials (n = 44)**

1. Playing tai chi helps prevent osteoporosis. Women's health at home and abroad. 2012(9):76.

2. Brooke-Wavell K, Duckham R, Carpenter H, et al. Higher bone mineral content at superior as well as inferior femoral neck in older adults habitually participating in multidirectional loading activities. Journal of Bone and Mineral Research. 2012;27.

3. Chen ZB, Hai WP, Deng XL. Effect of Taiji and basketball on bone metabolism in elderly people. Journal of Physical Education. 2002(06):55-6.

4. Dong RD. Research and Application of Scientific Fitness guidance system for Prevention of Postmenopausal Osteoporosis [Doctoral dissertation ]: Beijing Sport University 2005.

5. Ernst E. Favorable effect of Tai Chi in the prevention of osteoporosis. MMW-Fortschritte der Medizin. 2008;150(10):21.

6. Fan RY. Effect of physical exercise on bone mineral density of elderly women at high altitude. Qinghai Physical Education Science and Technology. 2021;62(1):3-7.

7. Fischer M, Woods NF, Kiel D, et al. Use of a pragmatic intervention embedded in a randomized trial to study longer-term impact of Tai Chi on multiple fall-related fracture risks in post-menopausal osteopenic women: Insights from qualitative interviews. Menopause. 2012;19(12):1387.

8. Gass R, Qin L, Au S, et al. Tai Chi Chuan and bone loss in postmenopausal women [2] (multiple letters). Archives of Physical Medicine and Rehabilitation. 2003;84(4):621-3.

9. Gui HL. Study on the effect of Taijiquan exercise on Fitness and Disease Prevention of the elderly and its Mechanism. Guizhou Sports Science and Techology. 2003(2):20-3.

10. Guo YJ, editor Study on the effect of Taijiquan exercise on Bone Metabolism Indexes in Postmenopausal Women. The first National Wushu Sports Conference and Wushu Science Conference; 2014; Tianjin, China.

11. Guo YJ, Jiang J, Xu HY. Effect of Taijiquan on bone metabolism in elderly women with type 2 diabetes mellitu. Contemporary Sports Technology. 2013;3(20):126-7.

12. He XQ, editor Effects of brisk walking on bone mineral density and blood lipids in middle-aged and elderly women. 2014 China Sports Physiology and Biochemistry Academic Conference; 2014; Guizhou, China.

13. Hong X, Lawson D. Effects of Tai Ji Exercise on Bone Structure and Function. Chinese Journal of Sports Medicine. 2005;24(1):63-8.

14. Huang ZQ. Effect of Social Danting and Taijiquan and Jogging on Bone Mineral Density and Body Composition of Women Aged from 40 to 65y Sichuan Sports Science. 2008(04):90-1+101.

15. Jing T, Lv HB, Chen HY, et al. Taijiquan exercise and low back Resistance training in the treatment of Primary Osteoporosis. Chinese Journal of Rehabilitation. 2007(06):435-6.

16. Li JY, Cheng L. Effect of different frequency of Taichi exercise on bone mineral density of older women. Chinese Journal of Osteoporosis. 2017;23(10):1309-12.

17. Lin F. The Effect of Long Time Taijiquan on Bone Density and Banlance Function of Menopause Women [Master Dissertation]: Shanghai University of Sport; 2010.

18. Liu JG, Zhou ZM. A Comparative Study of Shadow-boxing and Social Dancing on the Bone Mineral Density and Flexibility of the Elderly. Hubei Sports Science. 2012;31(03):325-6+30.

19. Ma ZY. Effect of Taijiquan exercise cycle on Lower limb function of Middle-aged and elderly Women. Chinese Journal of Gerontology. 2016;36(1):192-3.

20. Murphy L, Singh BB. Effects of 5-Form, Yang Style Tai Chi on older females who have or are at risk for developing osteoporosis. Physiotherapy Theory and Practice. 2008;24(5):311-20.

21. Pan YR, Wang MD, YUan JH, et al. Effect of Taichi Exercise on Bone Mineral Density in Natural Menopausal Women. Sichuan Sports Science. 2021;40(06):26-30+61.

22. Qi Q, Yu YY, Yu B, et al., editors. Effect of Taijiquan exercise on bone mineral density of postmenopausal women. The 8th Annual Conference of Chinese Rehabilitation Association; 2011; Chengdu, China.

23. Qie P, Li AQ, Zuo RW, et al. Effects of relaxation exercises such as Taijiquan on bone mineral density and microcirculation in middle-aged and elderly. Chinese Journal of Rehabilitation Medicine. 1995(04):175-6.

24. Qin L, Au S, Choy W, et al. Regular Tai Chi Chuan exercise may retard bone loss in postmenopausal women: A case-control study. Archives of Physical Medicine and Rehabilitation. 2002;83(10):1355-9.

25. Qin L, Choy W, Leung K, et al. Beneficial effects of regular Tai Chi exercise on musculoskeletal system. Journal of Bone and Mineral Metabolism. 2005;23(2):186-90.

26. Shen CL, Chyu MC, Yeh JK, et al. Green tea polyphenols and Tai Chi for bone health: Designing a placebo-controlled randomized trial. BMC Musculoskeletal Disorders. 2009;10(1):110.

27. Shen H. The Effect of Different Means of Fitness Exercises and Body Components on the Bone Density of Middle-Aged and Old Women. Journal of Chengdu Sport University. 2008;34(12):71-4.

28. Sun ZY. Effect of Taijiquan exercise on calcaneal bone mineral density and reaction time in middle-aged and elderly women. Contemporary Sports Technology. 2018;8(22):6-7.

29. Wang C, Yang Z, Chen Y. Effects of different exercises on the bone metabolism level of middle-aged and old women. Sheng Wu Yi Xue Gong Cheng Xue Za Zhi. 2009;26(6):1306-10.

30. Wang C, Yang ZH, editors. Effects of three forms of fitness exercise on bone mineral density and some blood indexes of middle-aged and elderly women in cities. Proceedings of the 8th China National Convention on Sport Sciences(Ⅰ); 2007; Beijing, China.

31. Wang J. Effect of exercise on bone mineral density of lumbar vertebrae L2-4 in postmenopausal women. Science & Technology of Stationery & Sporting Goods. 2014(10):173.

32. Wayne PM, Buring JE, Davis RB, et al. Tai Chi for osteopenic women: Design and rationale of a pragmatic randomized controlled trial. BMC Musculoskeletal Disorders. 2010;11:40.

33. Xiao L, Wang DX, Zhou Y. The effects of Taiji-Fan craftsmanship on aged postmenopausal women’s serum estrogen and bone metabolism index. Journal of Shaanxi Normal University(Natural Science Edition). 2009;37(05):105-8.

34. Xie BC, Chen SC, Wu JH, et al. The effects of Tai Chi exercise on bone mineral density in postmenopausal women. The Journal of sports medicine and physical fitness. 2019;59(6):1093-4.

35. Xu C. Tijiquan Exercise on Some Physiological Indices of Menopausal Women in the Changchun City [Master Dissertation]: Northeast Normal University; 2012.

36. Xu JW. Study on intervention Scheme for Prevention of Thoracolumbar fracture in Community Osteoporosis High-risk population. Chinese Journal of Osteoporosis. 2013;19(11):1180-3.

37. Xu JW, Yi LJ, Hu B, et al., editors. Study on intervention Scheme for Prevention of Thoracolumbar fracture in Community Osteoporosis High-risk population. Proceedings of the 6th Academic Congress of the Spinal Surgery of Chinese Assoc; 2013; Beijing, China.

38. Xuan L. The influence of different exercise methods on the Fitness efficacy of Middle-aged and elderly Women. Chinese Journal of Gerontology. 2018;38(24).

39. Yang HD, Gong CW. The effect of Taijiquan exercise on the balance ability of the elderly. Sports Research and Education. 2013;28(3):123-5.

40. Yu H. Influence of Taijiquan Exercise to Postmenopausal Women's Bone Mineral Density [Master Dissertation]. Chinese Doctoral Dissertations & Master's Theses Full-text Database (Master): Wuhan Institute of Physical Education 2006.

41. Yu YY, Qi Q, Yu B, et al. Effect of Taichi Exercise on Bone Mineral Density of Postmenopausal Women. Chinese Journal of Rehabilitation Theory and Practice. 2012;18(02):155-7.

42. Zhang Y, Zhao BL. Effects of regular Taijiquan exercise on body composition and bone mineral density of elderly intellectuals. Shaanxi Education. 2012(Z1):142-4.

43. Zhao L, Liu FP. Comparative Study on the Influence of Different Sports to Postmenopausal Women's Bone Metabolism. Fujian Sports Science and Technology. 2013;32(02):44-7.

44. Zou J, Lin F, Zhang L, et al. Effect of Long Time Taijiquan Training on Bone Density and Balance Function in Post-menopause Women. Chinese Journal of Rehabilitation Theory and Practice. 2011;17(01):80-2.

**Ineligible participants (n = 26)**

1. Bao QW, Gong C, Shen XZ, et al. Preventive effect of Taiji exercise on osteoporosis in elderly patients with type 2 diabetes mellitu. Chinese Journal of Gerontology. 2016;36(13):3246-8.

2. Du XX, Zhang MJ, Gou B, et al. Influence of Taiji Softball on Estrogen and Bone Metabolism Index of perimenopausal period Women. Journal of Xi'an Physical Education University. 2014;31(04):459-63.

3. Fan WY, Jin HY, Xu MH. Effect of auricular point pressing bean combined with Taijiquan exercise on pain and bone mineral density in elderly patients with osteoporosis. Chinese Journal of Traditional Medical Science and Technology. 2014;21(06):701-2.

4. Hsu WH, Hsu RWW, Lin ZR, et al. Effects of circuit exercise and Tai Chi on body composition in middle-aged and older women. Geriatrics and Gerontology International. 2015;15(3):282-8.

5. Hui SSC, Xie YJ, Woo J, et al. Effects of Tai Chi and Walking Exercises on Weight Loss, Metabolic Syndrome Parameters, and Bone Mineral Density: A Cluster Randomized Controlled Trial. Evidence-based Complementary and Alternative Medicine. 2015;2015:976123.

6. Li WQ, Huang XF, Bai M. Impact of Tai Chi exercise on the quality of life of women with osteoporosis in perimenopausal period Journal of Qilu nursing. 2010;16(11):16-7.

7. Liu W, Tong PJ, Xiao LW, et al. Oral application of Yigu Tang combined with shadow boxing exercises for treatment of osteoporosis with kidney—yang deficiency syndrome in the aged. The Journal of Traditional Chinese Orthopedics and Traumatology. 2018;30(11):6-12.

8. Luo Q, Xu GL, Chen P, et al. A preliminary approach to pre vent OCVF in patients with COPD complicated with oste oporosis. Chinese Journal of Osteoporosis. 2018;24(04):479-82.

9. Ma ZF. The Effect of Multi Band Spectrum Therapy Combined with Exercise Intervention on Osteoporosis [Master Dissertation]: Hebei Normal University 2020.

10. Ma ZY, Jia XF, Ding XL, et al. Study on the Effect of Core Muscle Strength Training Combined with TaiChi on Bone Mineral Density and Balance Function of the Elderly. Journal of Ningxia Medical University. 2020;42(08):791-4.

11. Shen CL, Williams JS, Chyu MC, et al. Comparison of the effects of Tai Chi and resistance training on bone metabolism in the elderly: A feasibility study. American Journal of Chinese Medicine. 2007;35(3):369-81.

12. Song Y. Effects of Taijiquan exercise on bone density and bone metabolism of primary osteoporosis sufferers. Journal of Physical Education. 2008(11):106-8.

13. Tang F, Xu JL, Liang HX. Intervention study on prevention of fracture in high-risk population of elderly patients with osteoporosis in a community of Shanghai. Chinese Community Doctors. 2018;34(35):159-60+63.

14. Wang TQ. Clinical Evaluation of Taijiquan combined with Qigu capsule in the Prevention and treatment of dyskinesia Syndrome in the elderly with liver and Kidney deficiency [Master Dissertation]: Shanghai University of Traditional Chinese Medicine; 2019.

15. Xia Q, Wang HB, Liu XQ. Clinical study on Prevention of falls in elderly patients with Osteoporosis. Chinese Journal of Physical Medicine and Rehabilitation. 2004(10):65.

16. Xiao C, Kang Y, Zhuang YC. Effects of Tai Chi Ball on Estrogen Levels, Bone Metabolism Index, and Muscle Strength of Perimenopausal Women. Journal of the American Geriatrics Society. 2015;63(12):2629-31.

17. Yang YF, Liu JH, Wu BH, et al. Effects of aerobic exercise combined with technetium [99Tc]-methylenediphosphonate on the bone density and the serum TRACP5b in elderly patients with osteoporosis

Anhui Medical and Pharmaceutical Journal. 2020;24(1).

18. Yang Z. Effects of Taiji Fitness Ball exercise on Bone Mineral density and Bone Metabolism of Lower extremities in the Middle-aged and elderly. New West. 2011(08):255+3.

19. Zhang S. A study on the effect of Taiji soft Ball on the physical Fitness of Middle-aged Women [Master Dissertation]: Central China Normal University; 2009.

20. Zhang WZ. Observations on the Effects of Tai Ji Quan with Caltrate D to Primary Osteoporosis [Master Dissertation]: Traditional Chinese Medicine University Of Guangzhou; 2011.

21. Zhao J. Effects of Tai Chi Chuan on the changes of bone mineral density of perimenopausal women. Chinese Journal of Tissue Engineering Research. 2020;24(02):176-80.

22. Zhao MY, Li N, Fan C, et al. About the influence of Tai Chi push exercise combined with nutrition intervention to recover of patients with primary osteoporosis. Liaoning Sport Science and Technology. 2015;37(03):44-6.

23. Zhou XJ, Liu CJ, Li J. On the Trend of Changes of bone mineral density of hyperlipidemia elderly By Taijiquan Exercise. Fujian Sports Science and Technology. 2014;33(02):39-42.

24. Zhou XY. Influence of Soft-ball Exercise on the Morphology and Bone Mineral Density in Elderly Female Body [Master Dissertation]: Xi'an Physical Education University; 2015.

25. Zhu YQ. Tai Chi and Whole-Body Vibration Therapy on Elderly:A Clinical Randomized Controlled Trial [Master Dissertation]: Chinese Pla General Hospital&Medical School; 2016.

26. Zhu YQ, Peng N. Tai chi and whole-body vibration therapy in the elderly: A randomized controlled trial. Journal of the American Geriatrics Society. 2016;64:S373.

**Ineligible interventions (n = 9)**

1. Chen C. Effect of Exercise Therapy on Bone Mineral Density, Lean Body Mass and Fat Mass of Postmenopausal Osteoporosis [Master Dissertation]: Nanjing University of Traditional Chinese Medicin; 2016.

2. Chen H. Exercise Therapy Has the Influence of Bone Mineral Density and Biochemical Index for Patients of Postmenopausal with Osteoporosis [Master Dissertation]: Nanjing University of Traditional Chinese Medicine 2015.

3. Chen YB, Xiong ZY, Zhou Y, et al. Study on Effect of Exercises Treatment and Exercises Prescription for Osteoporosis. Journal of Beijing Teachers College of Physical Education. 2002(04):38-40.

4. Deng SL. Study on Exercise Intervention Pattern and the Relationship between Physical Activity and Onset of Postmenopausal Osteoporosis [Doctoral dissertation]: Huazhong University of Science and Technology; 2008.

5. Guo HP, editor Intervention of Taijiquan exercise and Local Resistance training on Postmenopausal Osteoporosis. The 11th National Rehabilitation academic Conference of exercise Therapy Branch of Chinese Rehabilitation Medical Association; 2011; Shanghai, China.

6. Guo HP. Intervention of Taijiquan combined with local resistance training on postmenopausal osteoporosis [Master Dissertation]: Shanghai Jiaotong University; 2011.

7. Wang J. Influence of Taiji Softball to Postmenopausal Women's Bone Mineral Density and Bone Metabolism Index. Journal of Beijing Sport University. 2007(09):1226-8.

8. Wang J, Chen J, editors. Effects of different strength training programs on bone mineral density in postmenopausal women. Abstracts of the 11th National Convention on Sport Science of China; 2019; Nanjing, China.

9. Yao Y, Jiang YB, Sun YT, editors. Effect of Taiji soft ball exercise on calcaneal bone mineral density in elderly women. The third National Fitness Science Conference; 2014; Shenzheng, China.

**Irrelevant outcomes (n = 16)**

1. Alp A, Cansever S, Görgeç N, et al. Effects of Tai Chi exercise on functional and life quality assessments in senile osteoporosis. Turkiye Klinikleri Journal of Medical Sciences. 2009;29(3):687-95.

2. Chyu MC, James CR, Sawyer SF, et al. Effects of tai chi exercise on posturography, gait, physical function and quality of life in postmenopausal women with osteopaenia: a randomized clinical study. Clinical rehabilitation. 2010;24(12):1080-90.

3. Fischer M, Fugate-Woods N, Wayne PM. Use of pragmatic community-based interventions to enhance recruitment and adherence in a randomized trial of Tai Chi for women with osteopenia: Insights from a qualitative substudy. Menopause. 2014;21(11):1181-9.

4. Fu X. The Effect of Shadowboxing to the Physical Functions and Composition of Middle and Old People Journal of Gansu Normal Colleges. 2009;14(5):73-6.

5. Meng FL, editor Experimental study on the effect of Taijiquan exercise on improving the balance ability of female elderly with reduced bone mass. Abstracts of the 11th National Convention on Sport Science of China; 2019; Nanjing, China.

6. Meng FL. Experimental Study on the Effect of Taijiquan on Improving the Balance Ability of Elderly Women with Decreased Bone Mass [Master Dissertation]: Capital Institute of Physical Education; 2019.

7. Murphy L, Riley D, Rodgers J, et al. Effects of Tai Chi on Balance, Mobility, and Strength Among Older Persons Participating in an Osteoporosis Prevention and Education Program. Explore: The Journal of Science and Healing. 2005;1(3):192-3.

8. Patru S, Marcu IR, Bumbea AM, et al. Effects of tai chi exercise on muscle strength and balance in postmenopau-salwomen with osteopaenia. Osteoporosis International. 2017;28:S301.

9. Qian G, Xue K, Tang L, et al. Mitigation of Oxidative Damage by Green Tea Polyphenols and Tai Chi Exercise in Postmenopausal Women with Osteopenia. PLoS ONE. 2012;7(10).

10. Qin L, Cai YY, Qu SQ, et al., editors. A comparative study on the effects of Taijiquan and brisk walking on muscle strength and quality of life in climacteric women. 7th National Sports Science Congress; 2004; Beijing, China.

11. Shi D. Effect of traditional exercise training on bone mineral density and balance ability of elderly women [Master Dissertation]: Shanghai University of Traditional Chinese Medicine 2015.

12. Wang HR, Yu B, Lu YZ, et al. Effects of different kinds of taijiquan training on choice stepping reaction time in postmenopausal women. Chinese Journal of Rehabilitation Medicine. 2015;30(11):1135-9.

13. Wang J. Study on the effect of Taijiquan exercise on balance function of postoperative patients with breast cancer [Master Dissertation]: Tianjin Institute of Physical Education; 2018.

14. Wu FY, Shi XP, Chen JY. Effect of Tai Chi Exercises on Prevention of Osteoporotic Fracture in Elderly Women. Modern Hospital. 2016;16(11):1708-11.

15. Zhao J, Zhang L, Tian Y. Effect of 6 months of Tai Chi Chuan and calcium supplementation on bone health in females aged 50-59 years. Journal of exercise science and fitness. 2007;5(2):88‐94.

16. Zhu Y, Chen SY, Wu YP, et al. Effect of Tai Chi on the lumbar muscle strength in women with primary osteoporosis. Chinese Journal of Osteoporosis. 2014;20(01):45-8.

**Unavailable data (n = 4)**

1. Gao L, Liu D, Mao DW, et al., editors. A follow-up experimental study on the effects of Taijiquan and brisk walking exercise on bone mass in elderly women. Abstract of the 9th National Convention on Sport Science of China; 2011; Shanghai, China.

2. Leung PC, Qin L, Au SK. Prevention of osteoporotic fractures among high-risk groups of post-menopausal women. Hong kong medical journal. 2006;12(4):36‐9.

3. Li Q. Effects of Taijiquan, brisk walking and yoga on lower limb motor ability of postmenopausal women. Chinese Journal of Gerontology. 2016;36(10):2457-9.

4. Li YH, editor Effect of Taijiquan on bone mineral density and balance ability of middle-aged and elderly women. Chinese Anatomical Society Annual Meeting 2019; 2019; Yunnan, China.

**Duplicates (n = 8)**

1. Chen R, Li SC. Effects of Taiji and Fast-walking on the Bone Mineral Density and Metabolism in the Old Women Journal of Jilin Institute of Physical Education. 2011;27(01):87-8.

2. Fan LH. Experimental Study of Taijiquan and Brisk Walking Exercise Influence on the Physical Function of the Elderly People [Master Dissertation]: Shandong Physical Education Institute; 2013.

3. Gao Y. Experimental study on the effect of Taiji fan on bone mineral density in middle-aged and elderly women. Proceedings of 2010 Chinese Academic Conference on Sports Physiology and Biochemistry; Chengdu, China2010. p. 163-.

4. Shen CL, Chyu MC, Yeh JK, et al. Effect of green tea polyphenols and Tai Chi exercise on bone health in postmenopausal women with low bone mass: A 24-week placebo-controlled randomized trial. FASEB Journal. 2011;25.

5. Shen CL, Chyu MC, Yeh JK, et al. Effect of 24-week green tea polyphenols supplementation and Tai Chi exercise on bone biomarkers in postmenopausal osteopenic women. Journal of Bone and Mineral Research. 2010;25:S479.

6. Sun W, Wang JN, Yang CR, et al. Follow-up research on the effect of tai chi and brisk walking on bone mineral density and bone metabolism in elderly women. Chinese Journal of Osteoporosis. 2017;23(08):1034-40.

7. Xue Y, Hu Y, Wang O, et al. Effects of enhanced exercise and combined Vitamin D and calcium supplementation on muscle strength and fracture risk in postmenopausal Chinese women. Acta Academiae Medicinae Sinicae. 2017;39(3):345-51.

8. Zhou Y. Effects of Taiji hand exercise and calcium supplementation on bone mineral density in postmenopausal women. Bulletin of Sport Science & Technology. 2005(08):38.
