# Supplementary material for: The effect and safety of Tai Chi on bone health in postmenopausal women: A meta-analysis and trial sequential analysis
Source: Front Aging Neurosci. 2022 Sep 13;14:935326. doi: 10.3389/fnagi.2022.935326 (PMC9513206; doi:10.3389/fnagi.2022.935326)
Supplement: Supplementary file 4 [file Table_4.docx]

**Figure S1 The plots of sensitivity analysis for Tai Chi versus non-intervention on [bone](D:/software/%E6%9C%89%E9%81%93/Dict/Application/8.9.2.0/resultui/html/index.html#/javascript:;) [mineral](D:/software/%E6%9C%89%E9%81%93/Dict/Application/8.9.2.0/resultui/html/index.html#/javascript:;) [density](D:/software/%E6%9C%89%E9%81%93/Dict/Application/8.9.2.0/resultui/html/index.html#/javascript:;) (BMD)**

1. **BMD of lumbar spine**

**
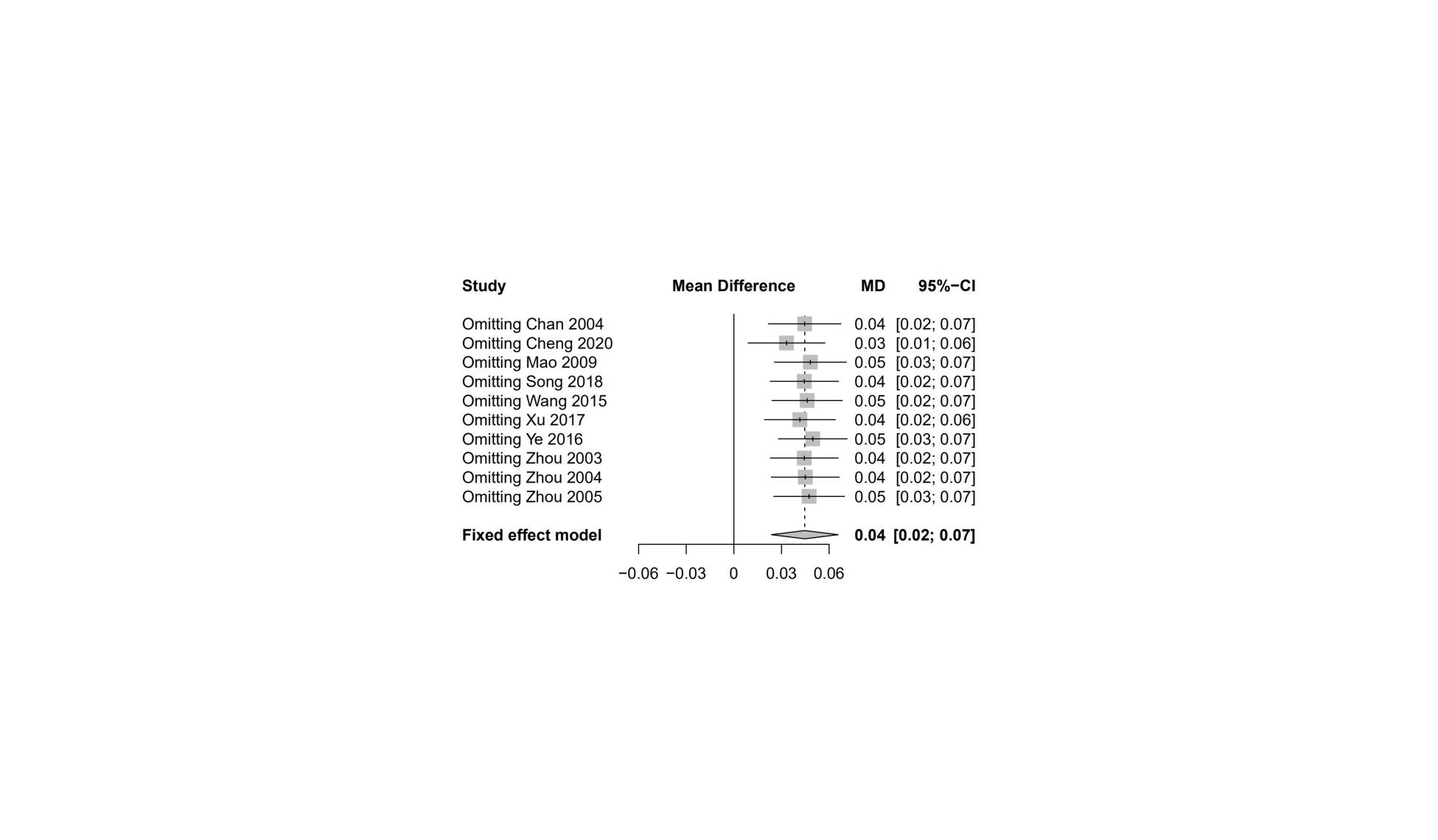
**

1. **BMD of femoral neck**

**
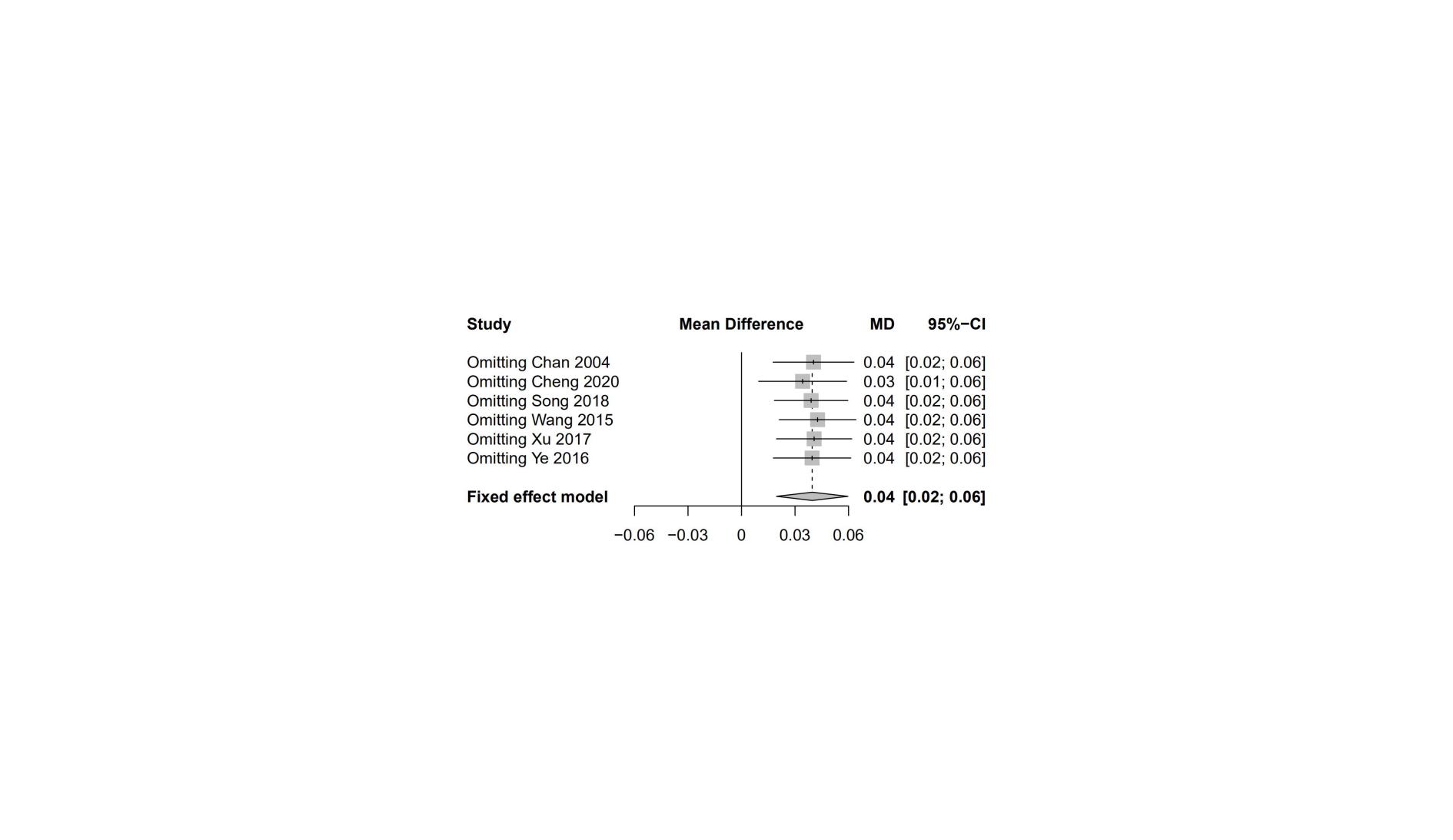
**

1. **BMD of Ward’s triangle**

**
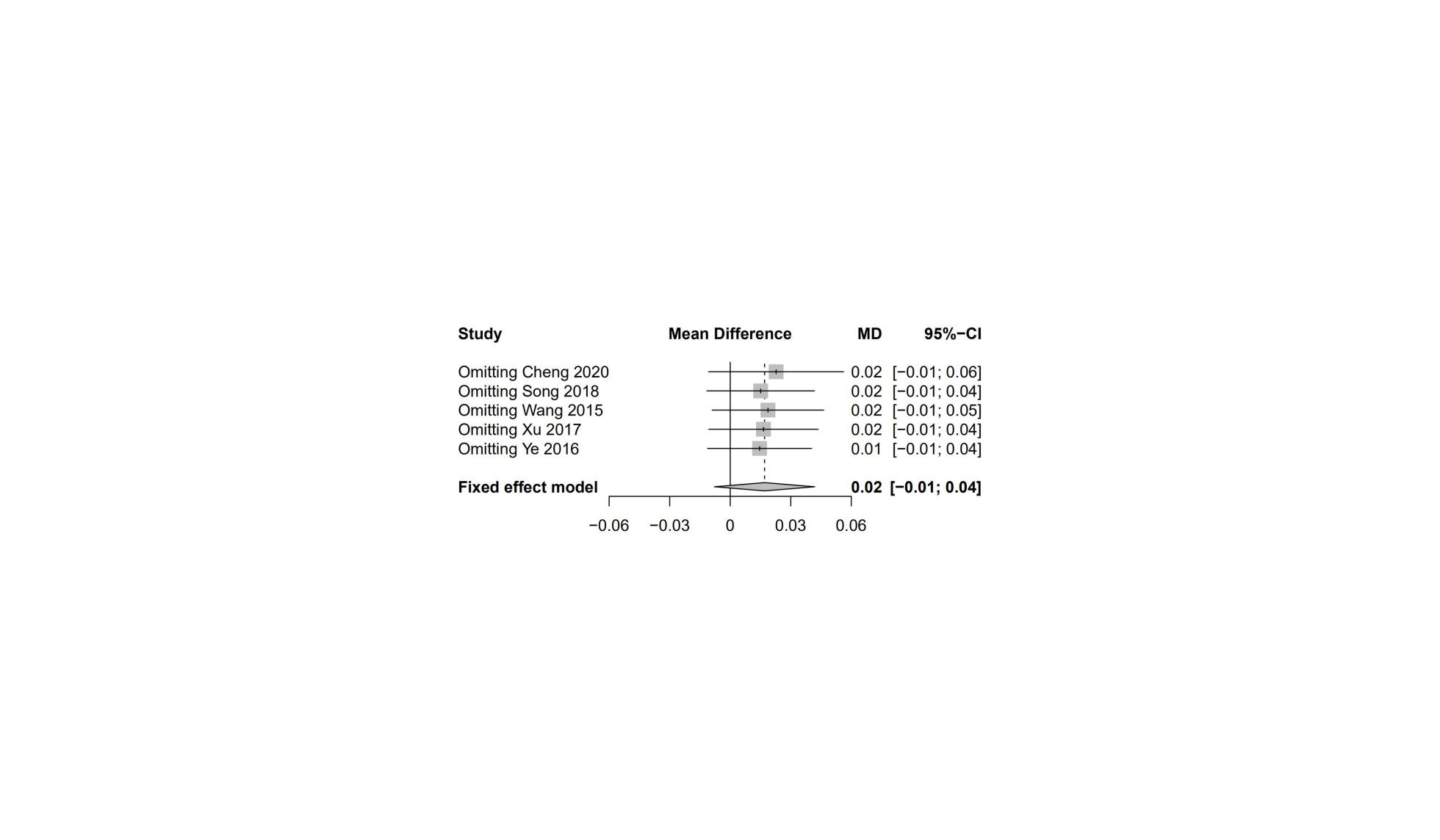
**

1. **BMD of trochanter**

**
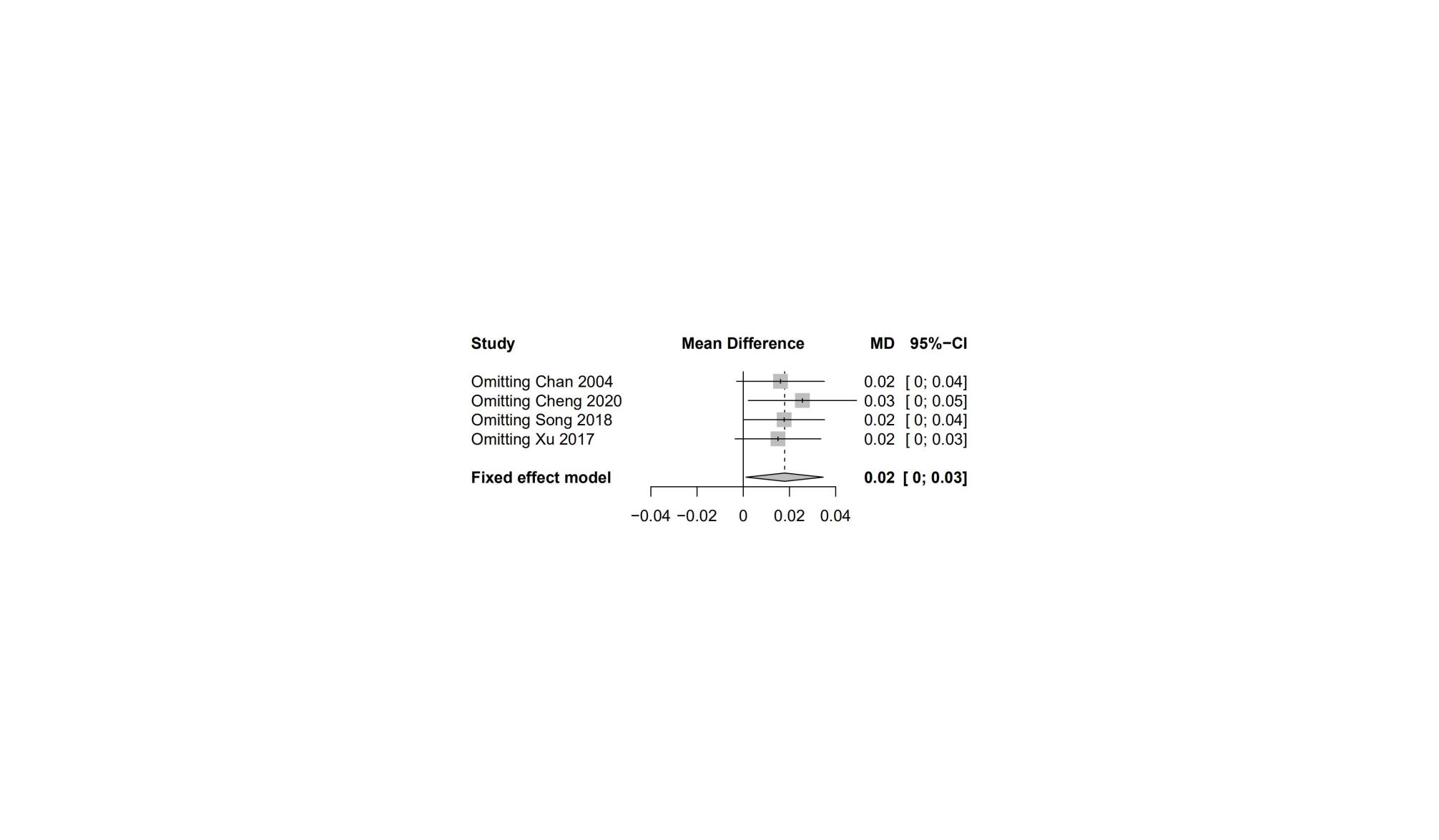
**

**Figure S2 The forest plots of Tai Chi versus non-intervention on** **percentage change of BMD**

**
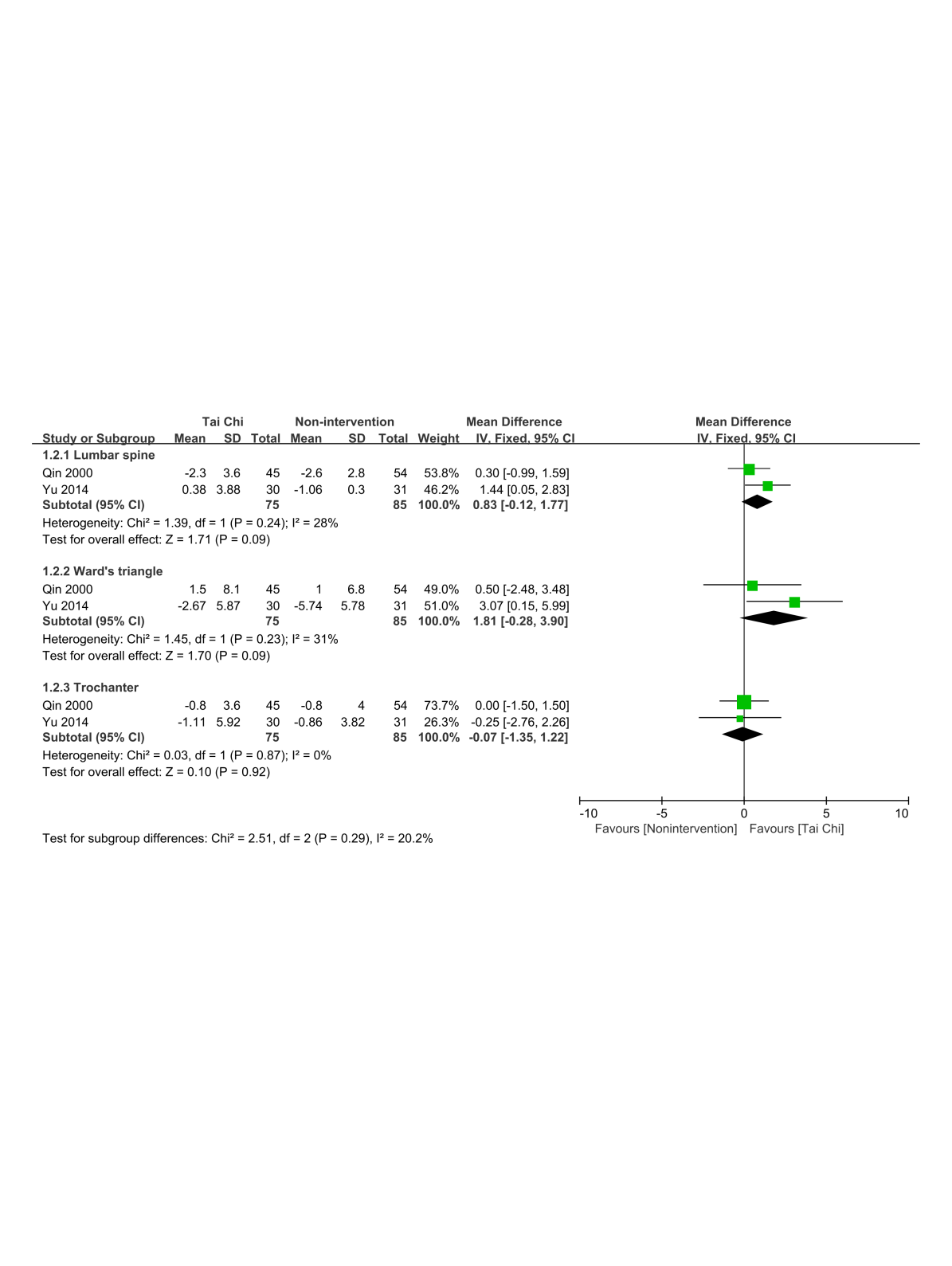
**

**Figure S3 The forest plot of sensitivity analysis for Tai Chi versus other exercises on BMD of lumbar spine**


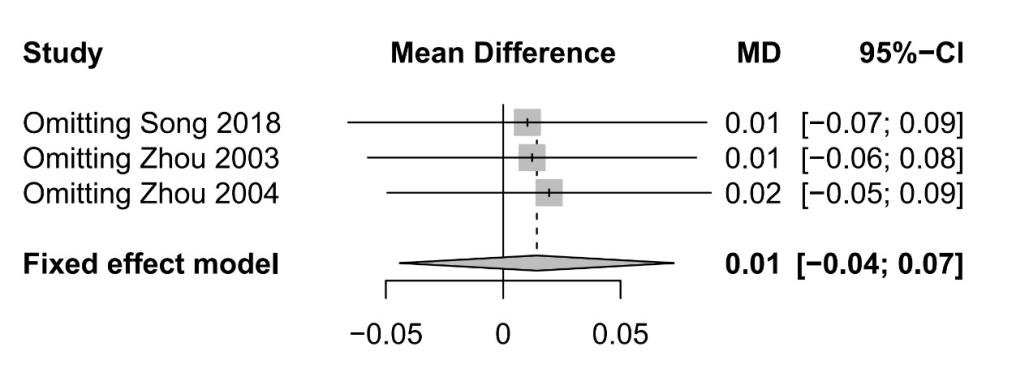


**Figure S4 The plots of sensitivity analysis for Tai Chi plus nutraceutical versus nutraceutical on BMD of lumbar spine**

**
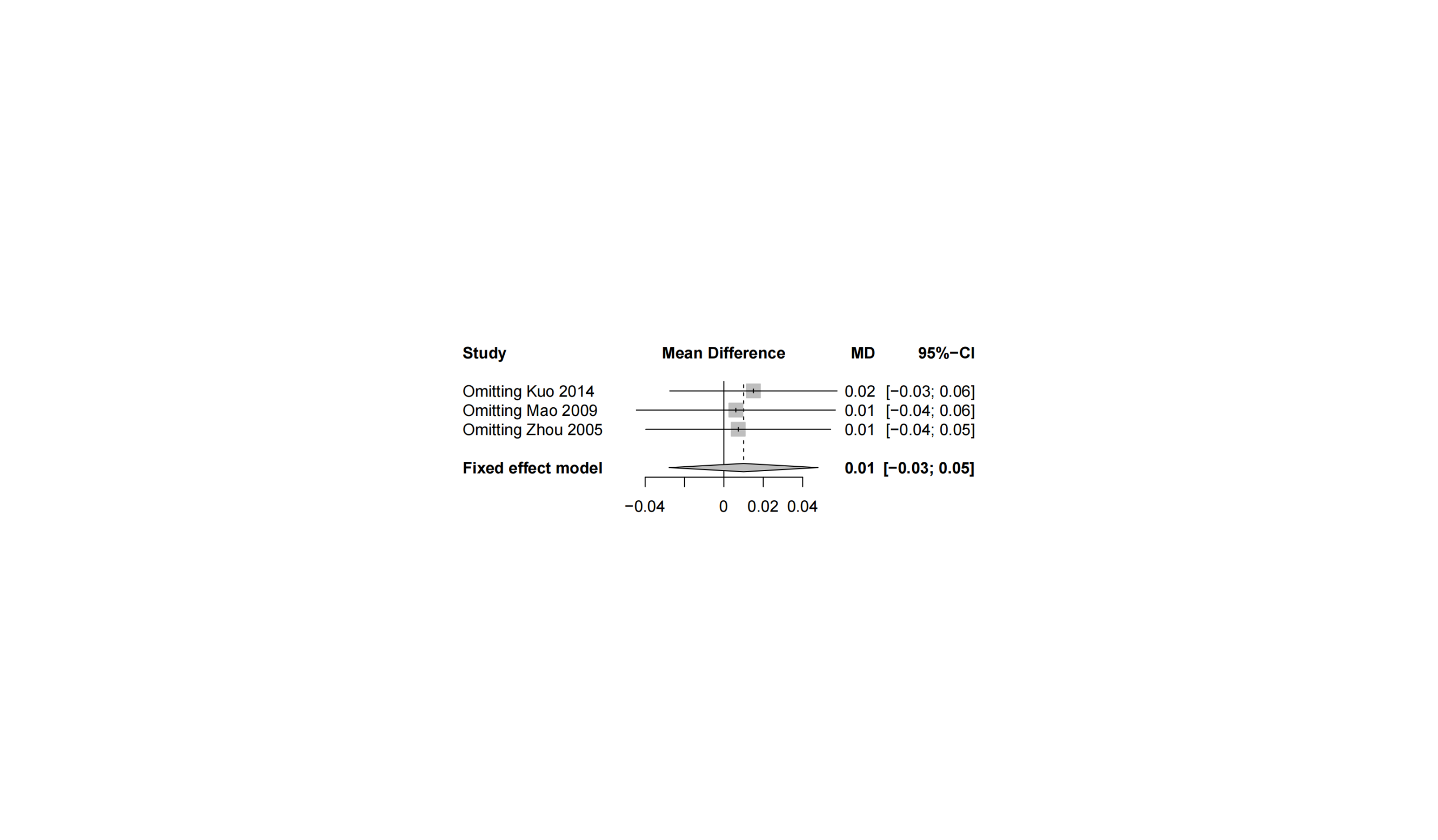
**

**Figure S5 The plots of sensitivity analysis for Tai Chi versus nonintervention on Calcaneus quantitative ultrasound**

1. **Speed of sound**

**
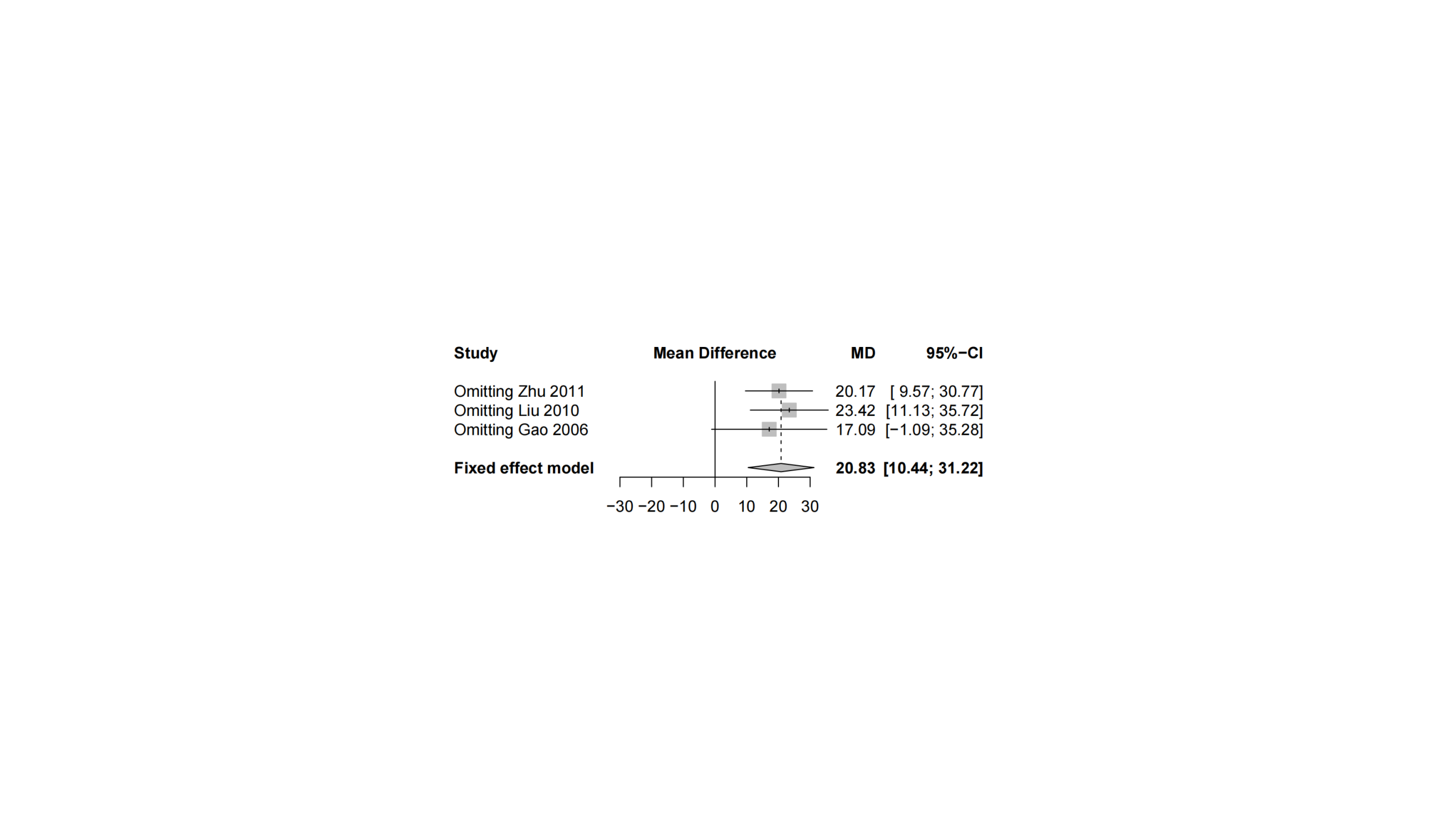
**

1. **BMD of calcaneus**

**
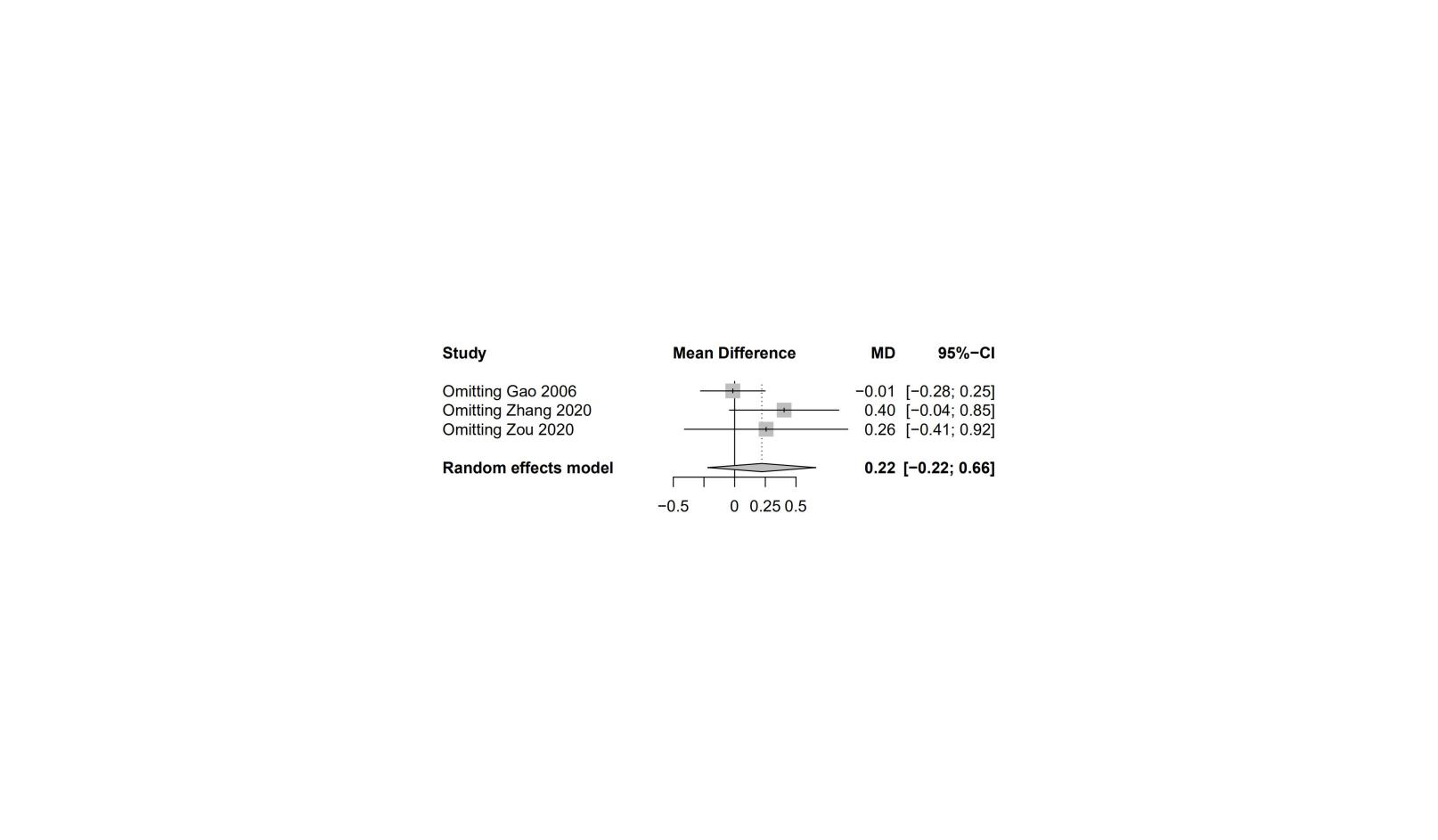
**

1. **Broadband ultrasonic attenuation**

**
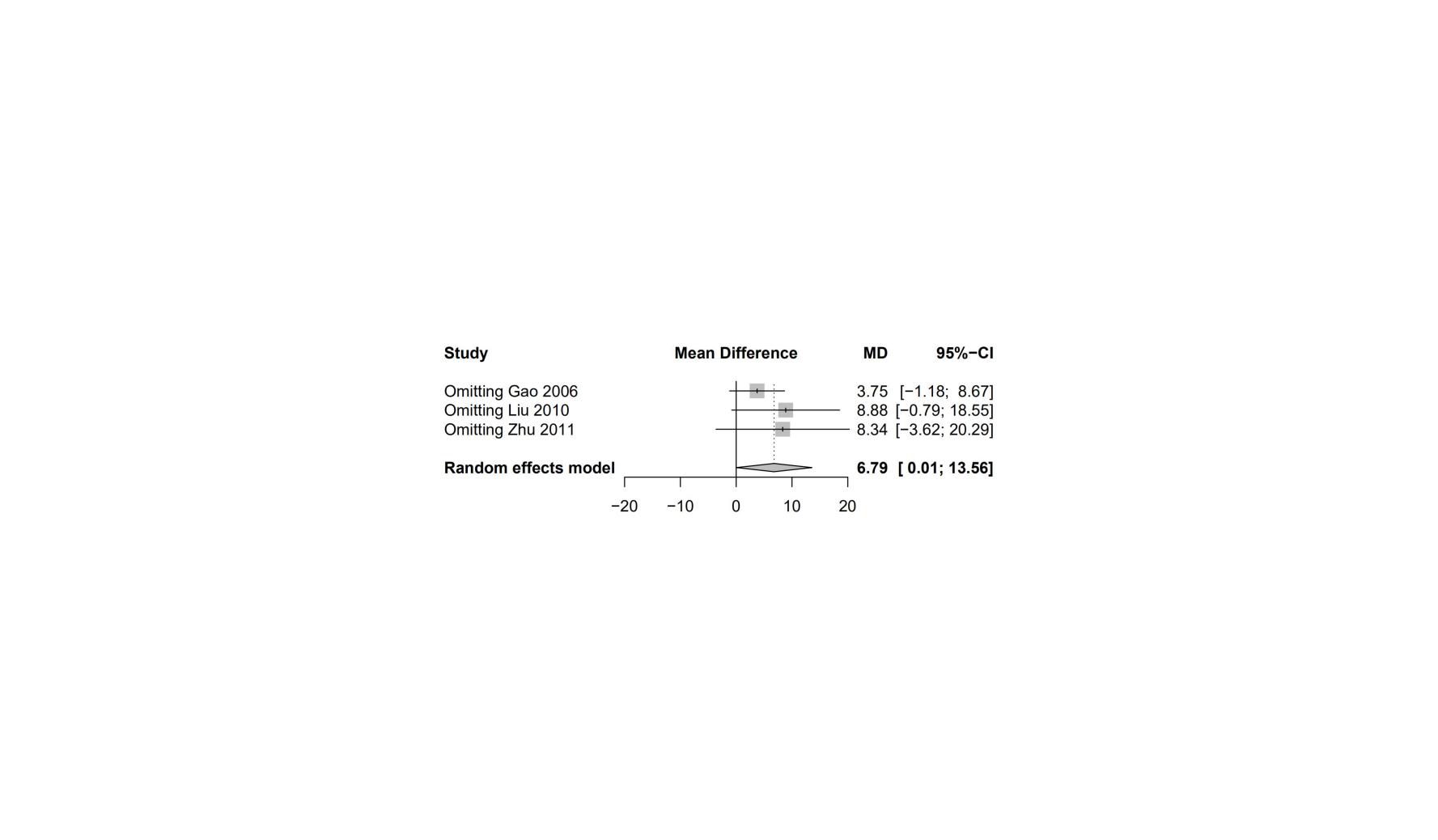
**

1. **Bone quality index**

**
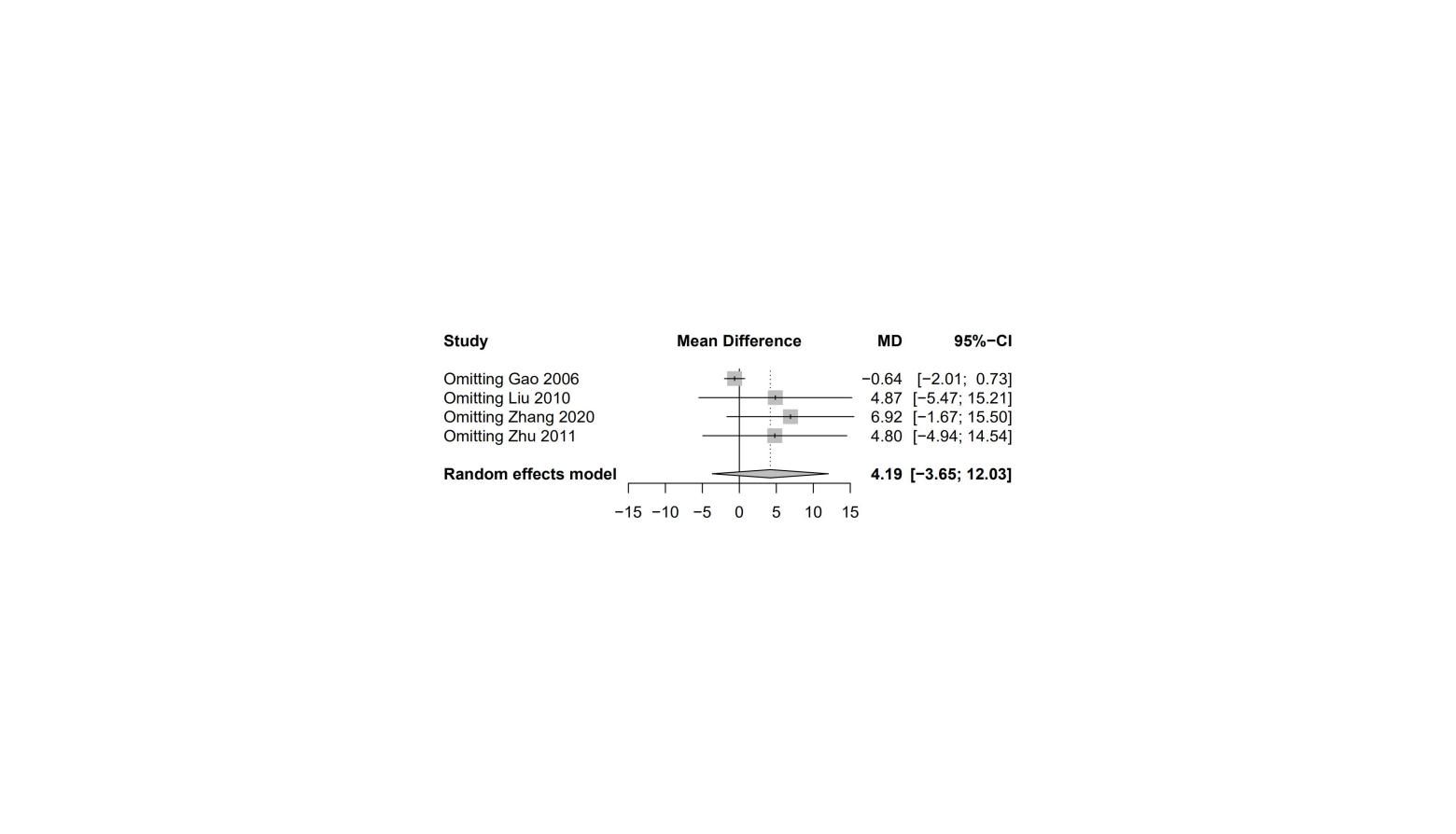
**

**Figure S6 The plots of sensitivity analysis for Tai Chi versus other exercises on calcaneus quantitative ultrasound**

1. **Speed of sound**

**
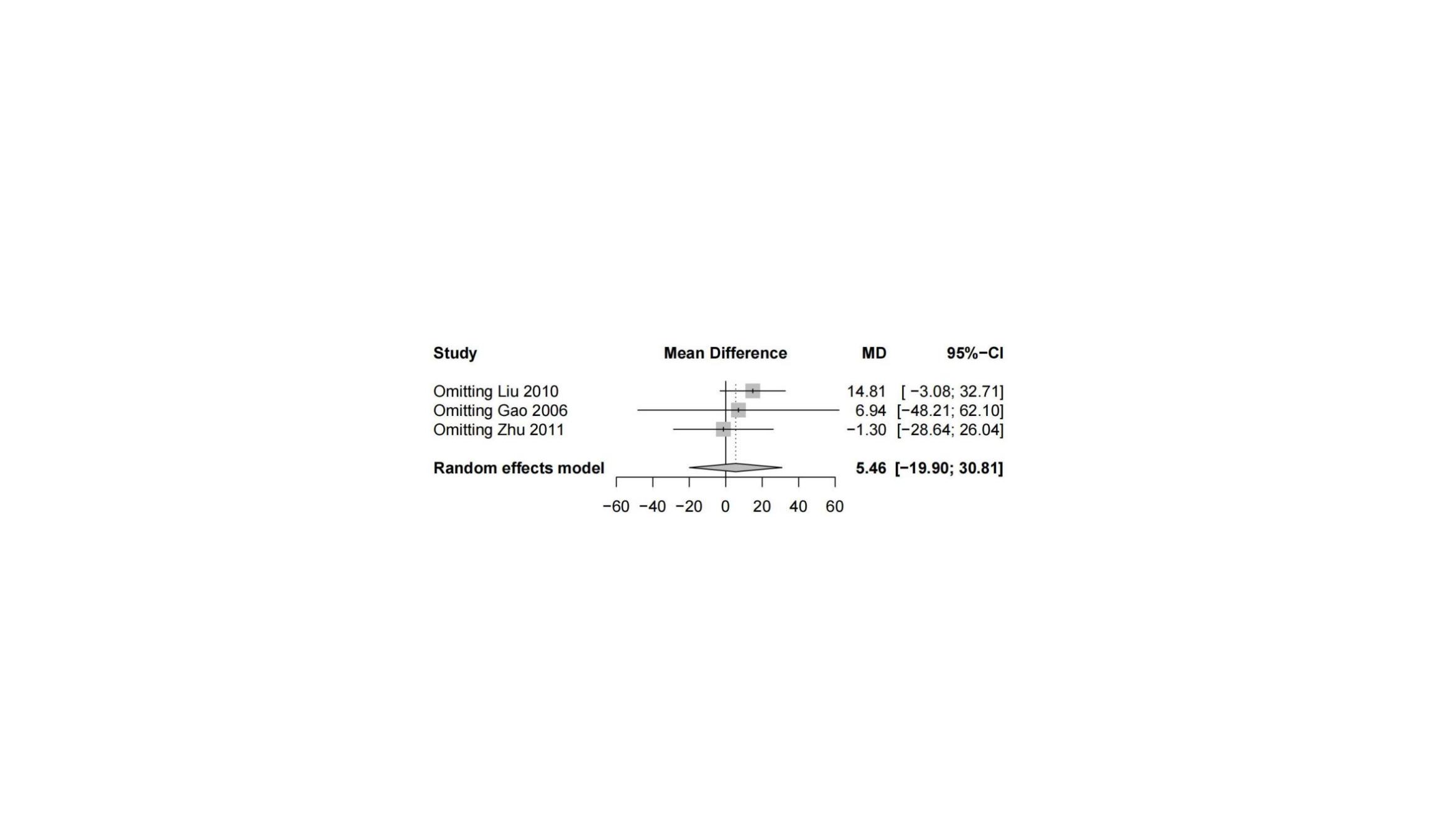
**

1. **BMD of calcaneus**

**
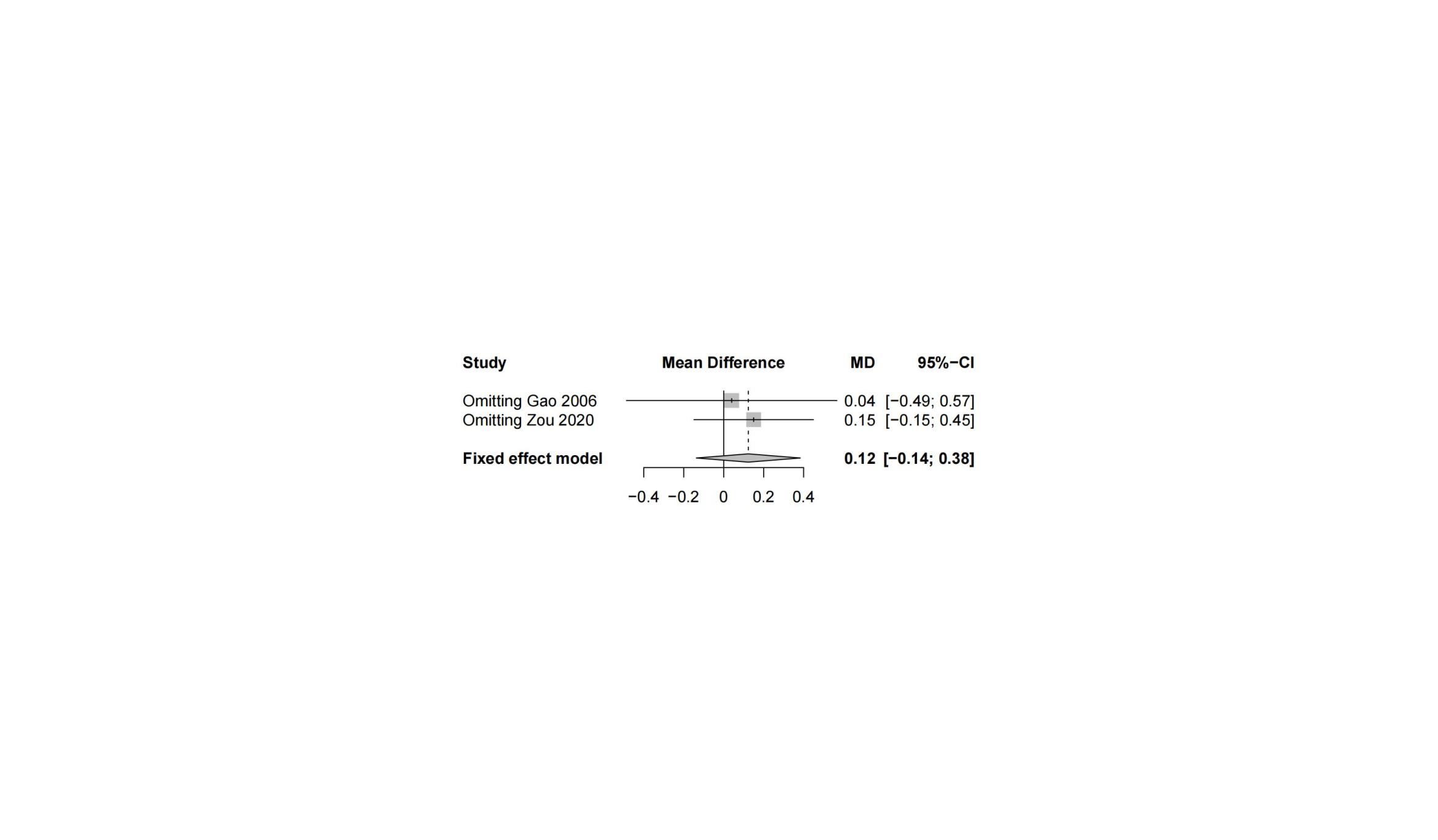
**

1. **Broadband ultrasonic attenuation**

**
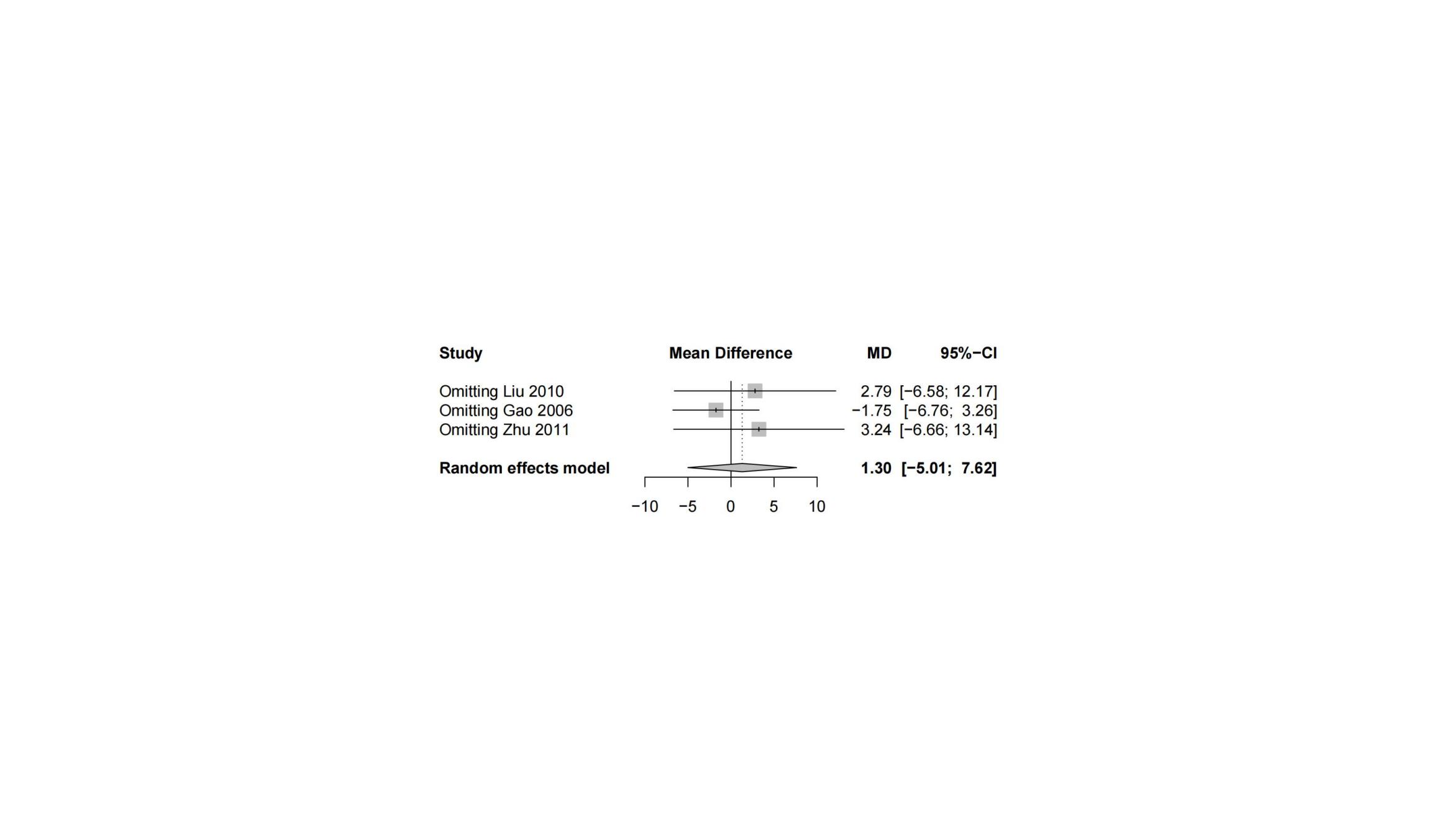
**

1. **Bone quality index**

**
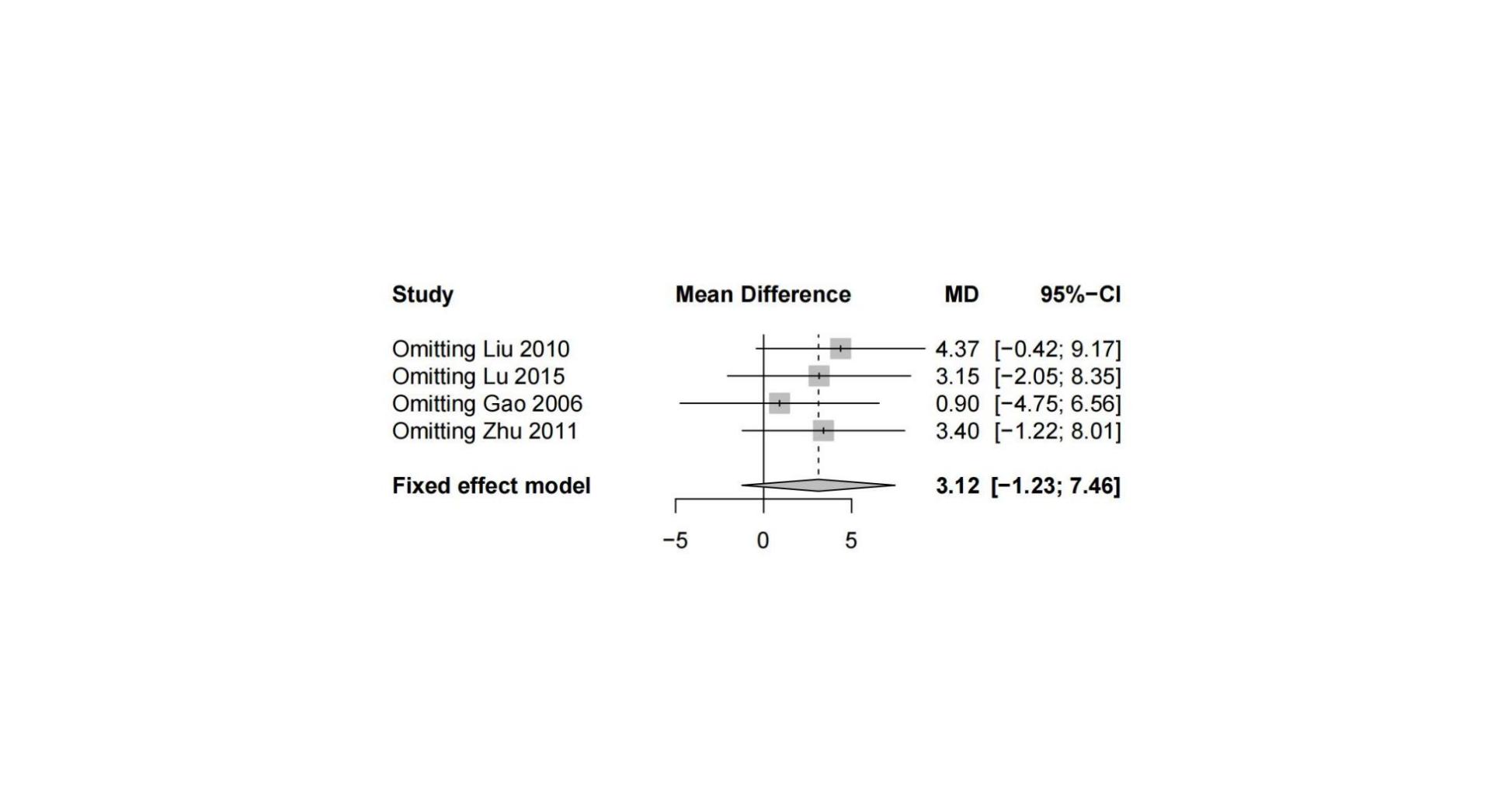
**

**Figure S7 The forest plots of subgroup analyses**

1. **BMD of lumbar spine**

**
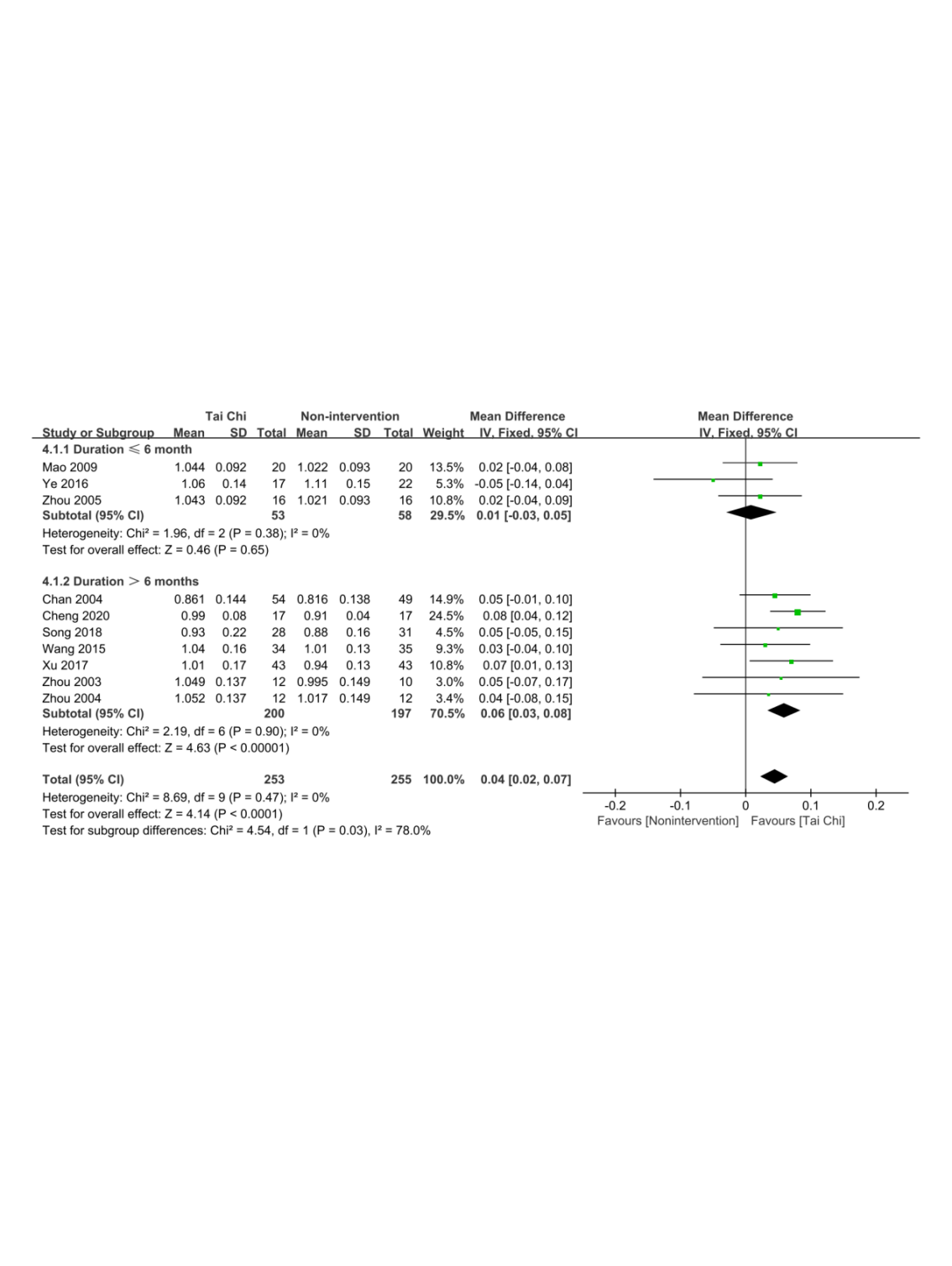
**

1. **BMD of femoral neck**

**
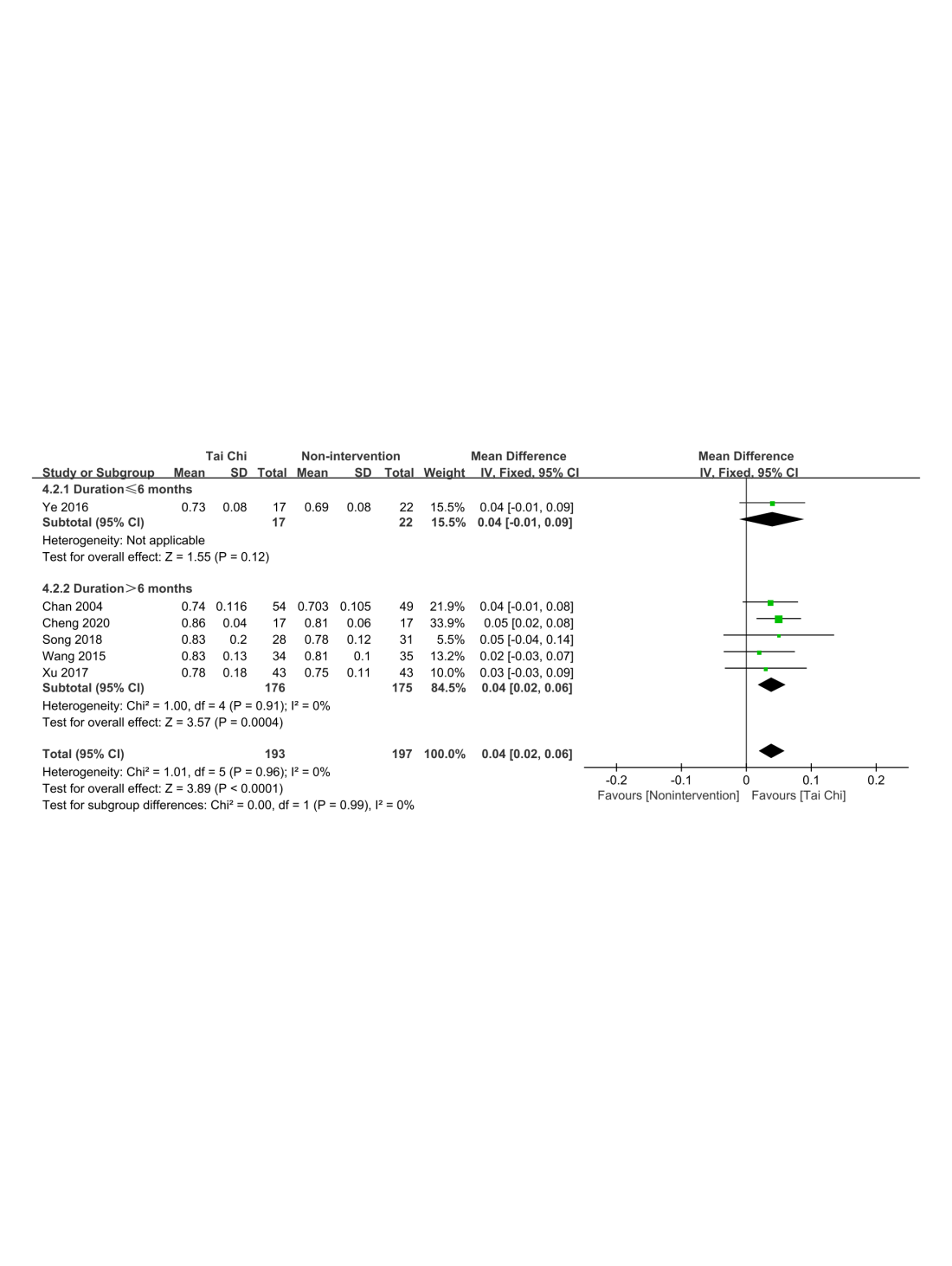
**

1. **BMD of Ward’s triangle**

**
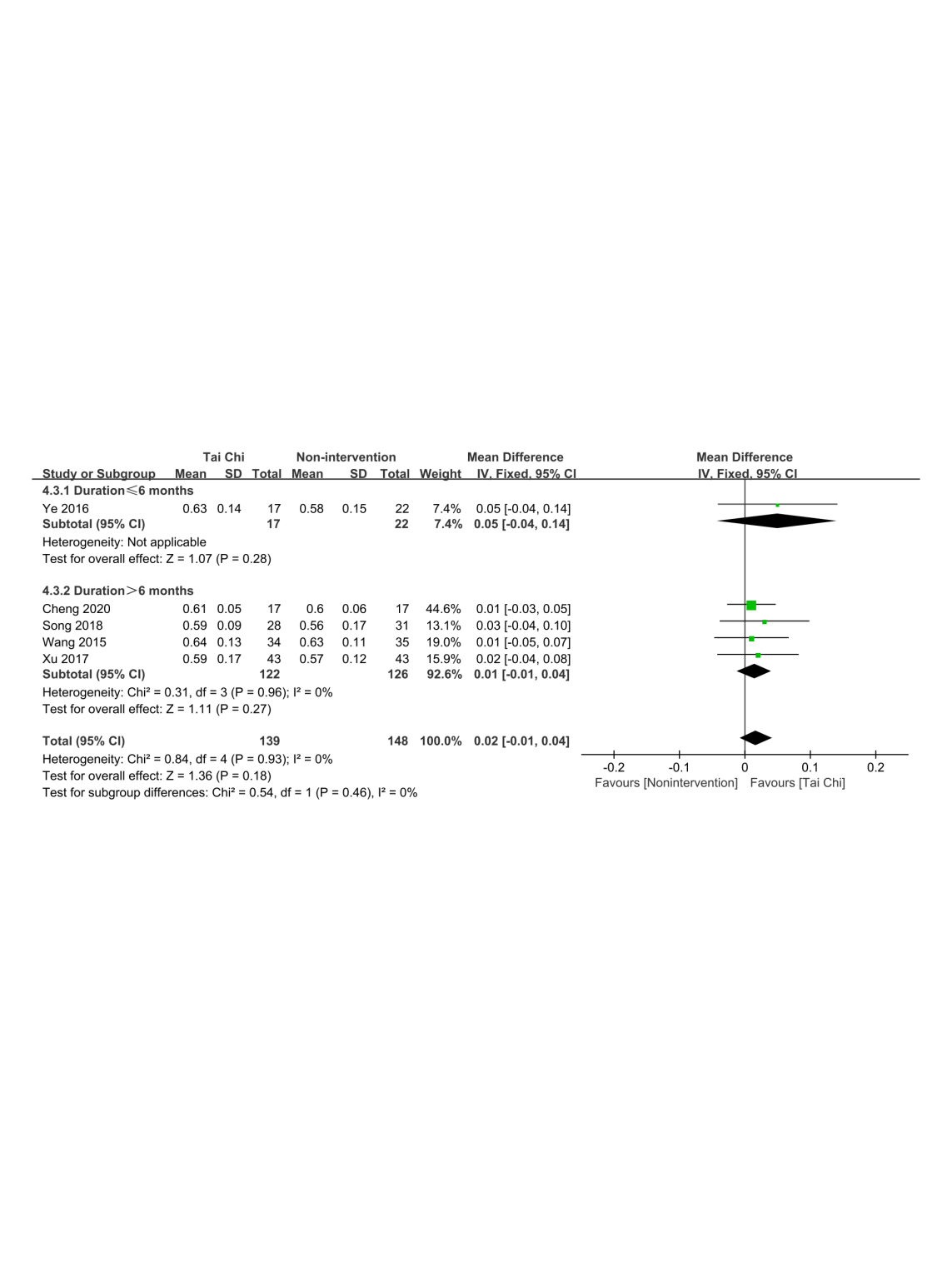
**

1. **BMD of trochanter**

**
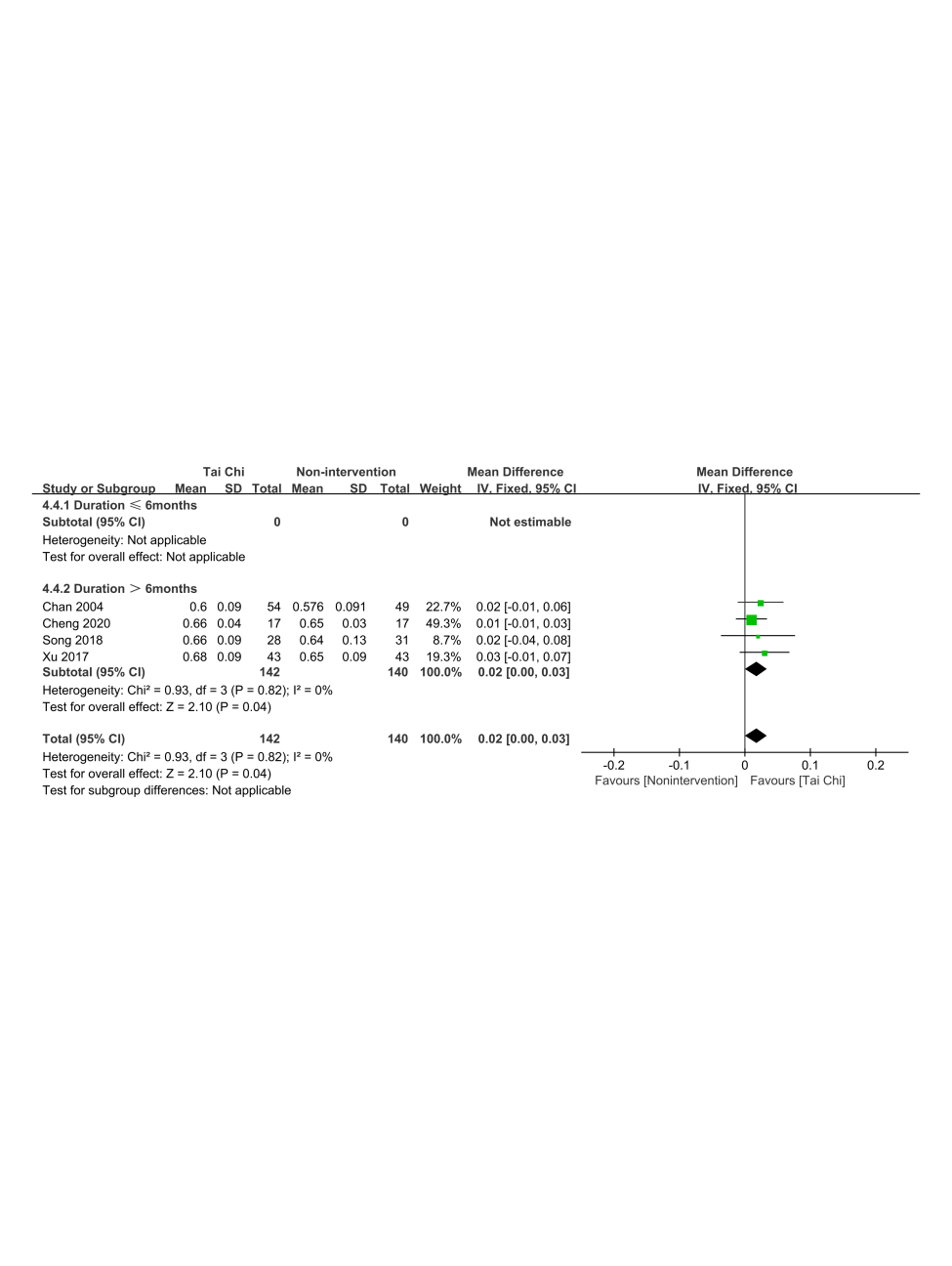
**
