# Supplementary material for: The effect and safety of Tai Chi on bone health in postmenopausal women: A meta-analysis and trial sequential analysis
Source: Front Aging Neurosci. 2022 Sep 13;14:935326. doi: 10.3389/fnagi.2022.935326 (PMC9513206; doi:10.3389/fnagi.2022.935326)

**The plots of trial sequential analysis of Tai Chi versus non-intervention.**

**(A) BMD of lumbar spine**


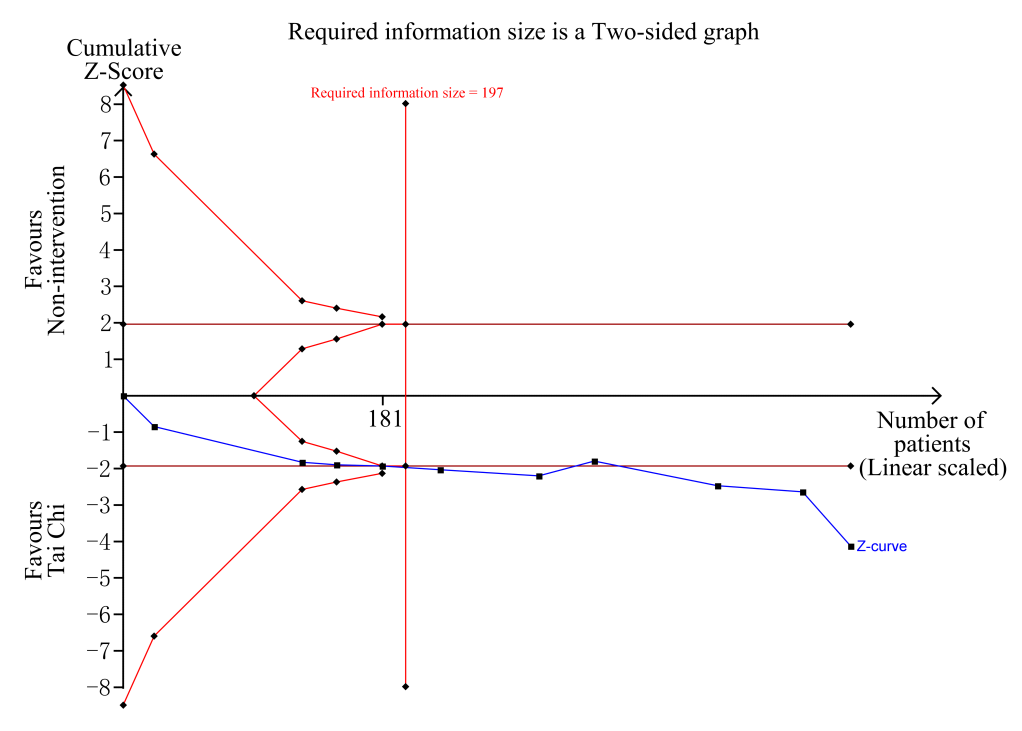


**(B) BMD of femoral neck**


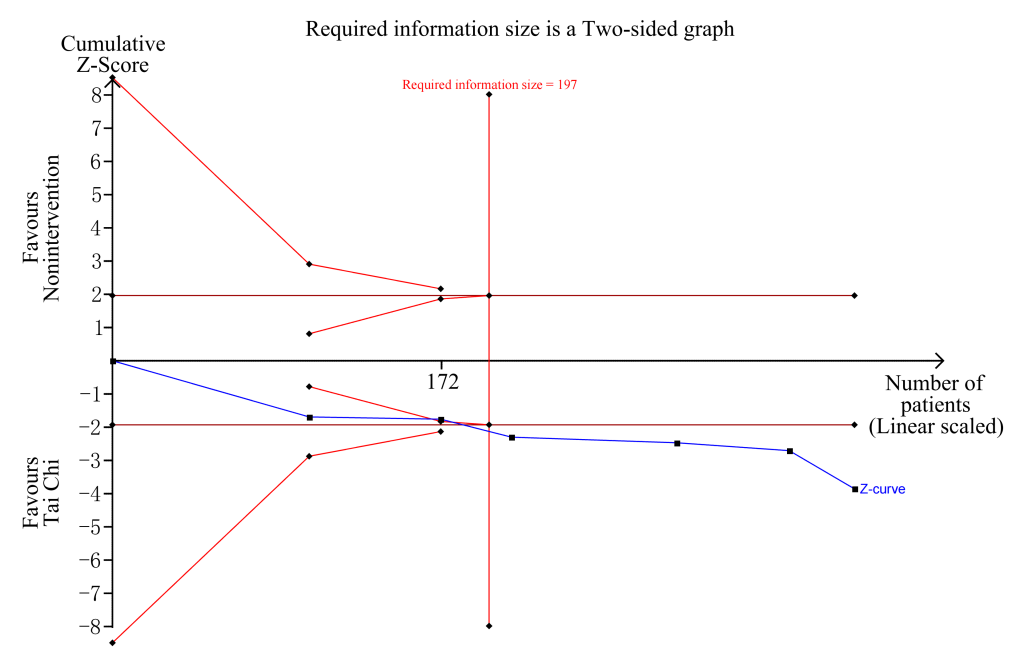


1. **BMD of Ward’s triangle**


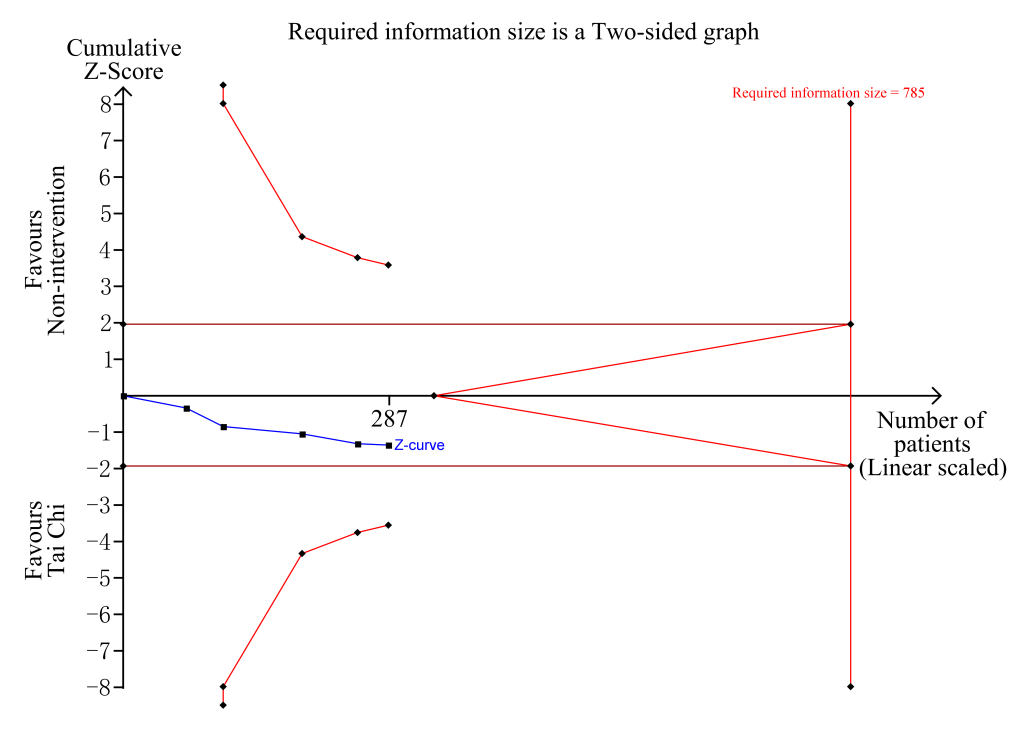


**(D) BMD of trochanter**


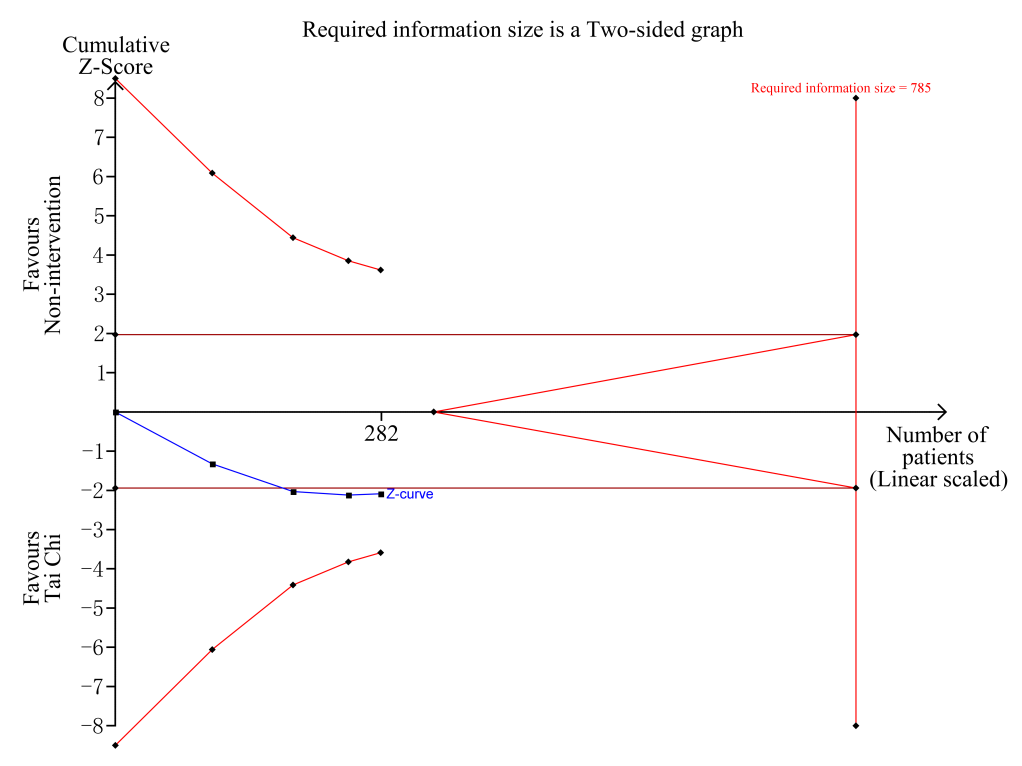

Supplement: Supplementary file 5 [file Table_5.docx]
