# Supplementary material for: The effect and safety of Tai Chi on bone health in postmenopausal women: A meta-analysis and trial sequential analysis
Source: Front Aging Neurosci. 2022 Sep 13;14:935326. doi: 10.3389/fnagi.2022.935326 (PMC9513206; doi:10.3389/fnagi.2022.935326)
Supplement: Supplementary file 6 [file Table_6.docx]

**Summary of findings 1. Tai Chi** **versus Non-intervention**

| **Tai Chi versus Non-intervention** | | | | | | |
| --- | --- | --- | --- | --- | --- | --- |
| **Patient or population:** Postmenopausal women  **Intervention:** Tai Chi **Comparison:** Non-intervention | | | | | | |
| **Outcomes** | **Illustrative comparative risks* (95% CI)** | | **Relative effect (95% CI)** | **No of Participants (studies)** | **Quality of the evidence (GRADE)** | **Comments** |
|  | Assumed risk | Corresponding risk |  |  |  |  |
|  | **Non-intervention** | **Tai Chi** |  |  |  |  |
| **BMD (Lumbar spine)** |  | The mean BMD (Lumbar spine) in the intervention groups was **0.04 higher** (0.02 to 0.07 higher) |  | 508 (10 studies) | ⊕⊕⊝⊝ **low** | Higher BMD indicates improvement |
| **BMD (Lumbar spine)**  **Duration of Tai Chi ≤ 6 months** |  | The mean BMD (Lumbar spine) when duration of Tai Chi ≤ 6 months in the intervention groups was **0.01 higher** (0.03 lower to 0.05 higher) |  | 111 (3 studies) | ⊕⊝⊝⊝ **very low** | Higher BMD indicates improvement |
| **BMD (Lumbar spine)**  **Duration of Tai Chi > 6 months** |  | The mean BMD (Lumbar spine) when duration of Tai Chi > 6 months in the intervention groups was **0.06 higher** (0.03 to 0.08 higher) |  | 397 (7 studies) | ⊕⊕⊝⊝ **low** | Higher BMD indicates improvement |
| **Percentage change of BMD (Lumbar spine)** |  | The mean percentage change of BMD (lumbar spine) in the intervention groups was **0.83 higher** (0.12 lower to 1.77 higher) |  | 160 (2 studies) | ⊕⊝⊝⊝ **very low**^1^ | Higher percentage change of BMD indicates improvement |
| **BMD (Femoral neck)** |  | The mean BMD (Femoral neck) in the intervention groups was **0.04 higher** (0.02 to 0.06 higher) |  | 390 (6 studies) | ⊕⊕⊝⊝ **low** | Higher BMD indicates improvement |
| **BMD (Femoral neck)**  **Duration of Tai Chi ≤ 6 months** |  | The mean BMD (Femoral neck) when duration of Tai Chi ≤ 6 months in the intervention groups was **0.04 higher** (0.01 lower to 0.09 higher) |  | 39 (1 study) | ⊕⊝⊝⊝ **very low** | Higher BMD indicates improvement |
| **BMD (Femoral neck)**  **Duration of Tai Chi > 6 months** |  | The mean BMD (Femoral neck) when duration of Tai Chi > 6 months in the intervention groups was **0.04 higher** (0.02 to 0.06 higher) |  | 351 (5 studies) | ⊕⊝⊝⊝ **very low** | Higher BMD indicates improvement |
| **BMD (Ward’s triangle)** |  | The mean BMD (Ward’s triangle) in the intervention groups was **0.02 higher** (0.01 lower to 0.04 higher) |  | 287 (5 studies) | ⊕⊝⊝⊝ **very low** | Higher BMD indicates improvement |
| **BMD (Ward’s triangle)**  **Duration of Tai Chi ≤ 6 months** |  | The mean BMD (Ward’s triangle) when duration of Tai Chi ≤6 months in the intervention groups was **0.05 higher** (0.04 lower to 0.14 higher) |  | 39 (1 study) | ⊕⊝⊝⊝ **very low** | Higher BMD indicates improvement |
| **BMD (Ward’s triangle)**  **Duration of Tai Chi** **> 6 months** |  | The mean BMD (Ward’s triangle) when duration of Tai Chi > 6 months in the intervention groups was **0.01 higher** (0.01 lower to 0.04 higher) |  | 248 (4 studies) | ⊕⊝⊝⊝ **very low** | Higher BMD indicates improvement |
| **Percentage change of BMD (Ward’s triangle)** |  | The mean percentage change of BMD (ward’s triangle) in the intervention groups was **1.81 higher** (0.28 lower to 3.90 higher) |  | 160 (2 studies) | ⊕⊝⊝⊝ **very low**^1^ | Higher percentage change of BMD indicates improvement |
| **BMD(Trochanter)** |  | The mean BMD (Trochanter) in the intervention groups was **0.02 higher** (0 to 0.03 higher) |  | 282 (4 studies) | ⊕⊝⊝⊝ **very low** | Higher BMD indicates improvement |
| **BMD(Trochanter)**  **Duration of Tai Chi > 6months** |  | The mean BMD (Trochanter) when duration of Tai Chi > 6months in the intervention groups was **0.02 higher** (0 to 0.03 higher) |  | 282 (4 studies) | ⊕⊝⊝⊝ **very low** | Higher BMD indicates improvement |
| **Percentage change of BMD (Trochanter)** |  | The mean percentage change of BMD (trochanter) in the intervention groups was **0.07 lower** (1.35 lower to 1.22 higher) |  | 160 (2 studies) | ⊕⊝⊝⊝ **very low**^1^ | Higher percentage change of BMD indicates improvement |
| **Percentage change of BMD (Total spine)** |  | The mean percentage change of BMD (Total spine) in the intervention groups was **0.88 lower** (2.23 lower to 0.47 higher) |  | 58 (1 study) | ⊕⊝⊝⊝ **very low**^1^ | Higher percentage change of BMD indicates improvement |
| **Percentage change of BMD (Total hip)** |  | The mean percentage change of BMD (Total hip) in the intervention groups was **2.32 higher** (0.60 to 4.04 higher) |  | 58 (1 study) | ⊕⊝⊝⊝ **very low**^1^ | Higher percentage change of BMD indicates improvement |
| **BMD of calcaneus** |  | The mean BMD of calcaneus in the intervention groups was **0.22 higher** (0.22 lower to 0.66 higher) |  | 87 (3 studies) | ⊕⊝⊝⊝ **very low** | Higher BMD indicates improvement |
| **Bone quality index** |  | The mean bone quality index in the intervention groups was **4.19 higher** (3.65 lower to 12.03 higher) |  | 115 (4 studies) | ⊕⊝⊝⊝ **very low** | Higher bone quality index indicates improvement |
| **Broadband ultrasonic attenuation** |  | The mean broadband ultrasonic attenuation in the intervention groups was **6.79 higher** (0.01 to 13.56 higher) |  | 78 (3 studies) | ⊕⊝⊝⊝ **very low** | Higher broadband ultrasonic attenuation indicates improvement |
| **Speed of sound** |  | The mean speed of sound in the intervention groups was **20.83 higher** (10.44 to 31.22 higher) |  | 78 (3 studies) | ⊕⊝⊝⊝ **very low** | Higher speed of sound indicates improvement |
| **Serum PINP** |  | The mean serum PINP in the intervention groups was **4.94 higher** (0.21 to 9.67 higher) |  | 283 (1 study) | ⊕⊝⊝⊝ **very low** | Higher PINP indicates improvement |
| **Serum CTX** |  | The mean serum CTX in the intervention groups was **0.02 higher** (0.02 lower to 0.06 higher) |  | 283 (1 study) | ⊕⊝⊝⊝ **very low** | Lower CTX indicates improvement |
| **Serum ALP** |  | The mean serum ALP in the intervention groups was **3.84 higher** (12.26 lower to 19.94 higher) |  | 30 (1 study) | ⊕⊝⊝⊝ **very low** | Higher ALP indicates improvement |
| *The basis for the **assumed risk** (e.g. the median control group risk across studies) is provided in footnotes. The **corresponding risk** (and its 95% confidence interval) is based on the assumed risk in the comparison group and the **relative effect** of the intervention (and its 95% CI). **CI:** Confidence interval; **PINP:** Procollagen type I N-terminal propeptide; **CTX:** C-terminal telopeptide of type I collagen; **ALP**: alkaline phosphatase. | | | | | | |
| GRADE Working Group grades of evidence **High quality:** Further research is very unlikely to change our confidence in the estimate of effect.  **Moderate quality:** Further research is likely to have an important impact on our confidence in the estimate of effect and may change the estimate. **Low quality:** Further research is very likely to have an important impact on our confidence in the estimate of effect and is likely to change the estimate. **Very low quality:** We are very uncertain about the estimate. | | | | | | |

**Summary of findings 2. Tai Chi versus Other exercises**

| **Tai Chi versus Other exercises** | | | | | | |
| --- | --- | --- | --- | --- | --- | --- |
| **Patient or population:** Postmenopausal women **Intervention:** Tai Chi **Comparison:** Other exercises | | | | | | |
| **Outcomes** | **Illustrative comparative risks* (95% CI)** | | **Relative effect (95% CI)** | **No of Participants (studies)** | **Quality of the evidence (GRADE)** | **Comments** |
|  | Assumed risk | Corresponding risk |  |  |  |  |
|  | **Other exercises** | **Tai Chi** |  |  |  |  |
| **BMD (Lumbar spine)** |  | The mean BMD (Lumbar spine) in the intervention groups was **0.01 higher** (0.04 lower to 0.07 higher) |  | 105 (3 studies) | ⊕⊝⊝⊝ **very low** | Higher BMD indicates improvement |
| **BMD (Femoral neck)** |  | The mean BMD (Femoral neck) in the intervention groups was **0.2 Higher** (0.07 lower to 0.11 higher) |  | 57 (1 study) | ⊕⊝⊝⊝ **very low** | Higher BMD indicates improvement |
| **BMD (Ward’s triangle)** |  | The mean BMD (Ward’s triangle) in the intervention groups was **0.01 lower** (0.08 lower to 0.06 higher) |  | 57 (1 study) | ⊕⊝⊝⊝ **very low** | Higher BMD indicates improvement |
| **BMD(Trochanter)** |  | The mean BMD (Trochanter) in the intervention groups was **0.01 higher** (0.03 lower to 0.05 higher) |  | 57 (1 study) | ⊕⊝⊝⊝ **very low** | Higher BMD indicates improvement |
| **Percentage change of BMD (Total spine)** |  | The mean percentage change of BMD (Total spine) in the intervention groups was **1.88 lower** (3.24 to 0.52 lower) |  | 58 (1 study) | ⊕⊝⊝⊝ **very low** | Higher percentage change of BMD indicates improvement |
| **Percentage change of BMD (Total hip)** |  | The mean percentage change of BMD (Total hip) in the intervention groups was 0.02 **lower** (1.59 lower to 1.55 higher) |  | 58 (1 study) | ⊕⊝⊝⊝ **very low** | Higher percentage change of BMD indicates improvement |
| **BMD of calcaneus** |  | The mean BMD of calcaneus in the intervention groups was **0.12 higher** (0.14 lower to 0.38 higher) |  | 52 (2 studies) | ⊕⊝⊝⊝ **very low** | Higher BMD indicates improvement |
| **Bone quality index** |  | The mean bone quality index in the intervention groups was **3.12 higher** (1.23 lower to 7.46 higher) |  | 140 (4 studies) | ⊕⊝⊝⊝ **very low** | Higher bone quality index indicates improvement |
| **Broadband ultrasonic attenuation** |  | The mean broadband ultrasonic attenuation in the intervention groups was **1.30 lower** (5.01 lower to 7.62 higher) |  | 79 (3 studies) | ⊕⊝⊝⊝ **very low** | Higher broadband ultrasonic attenuation indicates improvement |
| **Speed of sound** |  | The mean speed of sound in the intervention groups was **5.46 higher** (19.90 lower to 30.81 higher) |  | 79 (3 studies) | ⊕⊝⊝⊝ **very low** | Higher speed of sound indicates improvement |
| **Serum ALP** |  | The mean serum ALP in the intervention groups was **2.65 lower** (15.99 lower to 10.69 higher) |  | 31 (1 study) | ⊕⊝⊝⊝ **very low** | Higher ALP indicates improvement |
| *The basis for the **assumed risk** (e.g. the median control group risk across studies) is provided in footnotes. The **corresponding risk** (and its 95% confidence interval) is based on the assumed risk in the comparison group and the **relative effect** of the intervention (and its 95% CI).  **CI:** Confidence interval; **ALP:** alkaline phosphatase. | | | | | | |
| GRADE Working Group grades of evidence **High quality:** Further research is very unlikely to change our confidence in the estimate of effect.  **Moderate quality:** Further research is likely to have an important impact on our confidence in the estimate of effect and may change the estimate. **Low quality:** Further research is very likely to have an important impact on our confidence in the estimate of effect and is likely to change the estimate. **Very low quality:** We are very uncertain about the estimate. | | | | | | |

**Summary of findings 3. Tai Chi plus nutraceutical versus nutraceutical**

| **Tai Chi plus nutraceutical versus nutraceutical** | | | | | | |
| --- | --- | --- | --- | --- | --- | --- |
| **Patient or population:** Postmenopausal women **Intervention:** Tai Chi plus nutraceutical **Comparison:** Nutraceutical | | | | | | |
| **Outcomes** | **Illustrative comparative risks* (95% CI)** | | **Relative effect (95% CI)** | **No of Participants (studies)** | **Quality of the evidence (GRADE)** | **Comments** |
|  | Assumed risk | Corresponding risk |  |  |  |  |
|  | **Nutraceutical** | **Tai Chi plus nutraceutical** |  |  |  |  |
| **BMD (Lumbar spine)** |  | The mean BMD (Lumbar spine) in the intervention groups was **0.01 higher** (0.03 lower to 0.05 higher) |  | 113 (3 studies) | ⊕⊝⊝⊝ **very low** | Higher BMD indicates improvement |
| **BMD (Femoral neck)** |  | The mean BMD (Femoral neck) in the intervention groups was **0.03 lower** (0.10 lower to 0.04 higher) |  | 41 (1 study) | ⊕⊝⊝⊝ **very low** | Higher BMD indicates improvement |
| **BMD (Total hip) (Tai Chi plus Standard care vs. Standard care)** |  | The mean BMD (Femoral neck) in the intervention groups was **0.00 lower** (0.04 lower to 0.03 higher) |  | 86 (1 study) | ⊕⊝⊝⊝ **very low** | Higher Percentage change of BMD indicates improvement |
| **Serum ALP** |  | The mean serum ALP in the intervention groups was **8.6 higher** (0.55 lower to 17.75 higher) |  | 74 (1 study) | ⊕⊝⊝⊝ **very low** | Higher ALP indicates improvement |
| **Serum TRAP** |  | The mean serum TRAP in the intervention groups was **0.15 lower** (0.72 lower to 0.42 higher) |  | 74 (1 study) | ⊕⊝⊝⊝ **very low** | Lower TRAP indicates improvement |
| **Serum BAP** |  | The mean serum BAP in the intervention groups was **1.5 higher** (3.08 lower to 6.08 higher) |  | 74 (1 study) | ⊕⊝⊝⊝ **very low** | Higher BAP indicates improvement |
| **Serum CTX (Tai Chi plus Standard care vs. Standard care)** |  | The mean serum CTX in the intervention groups was **0.06 lower** (0.18 lower to 0.06 higher) |  | 86 (1 study) | ⊕⊝⊝⊝ **very low** | Lower CTX indicates improvement |
| **Serum OSC (Tai Chi plus Standard care vs. Standard care)** |  | The mean serum OSC in the intervention groups was **0.12 lower** (2.36 lower to 2.12 higher) |  | 86 (1 study) | ⊕⊝⊝⊝ **very low** | Higher OSC indicates improvement |
| *The basis for the **assumed risk** (e.g. the median control group risk across studies) is provided in footnotes. The **corresponding risk** (and its 95% confidence interval) is based on the assumed risk in the comparison group and the **relative effect** of the intervention (and its 95% CI). **CI:** Confidence interval; **CTX:** C-terminal telopeptide of type I collagen; **ALP**: alkaline phosphatase.; **TRAP:** Tartrate-resistant acid phosphatase; **BAP:** Bone-specific alkaline phosphatase; **OSC:** Osteocalcin | | | | | | |
| GRADE Working Group grades of evidence **High quality:** Further research is very unlikely to change our confidence in the estimate of effect.  **Moderate quality:** Further research is likely to have an important impact on our confidence in the estimate of effect and may change the estimate. **Low quality:** Further research is very likely to have an important impact on our confidence in the estimate of effect and is likely to change the estimate. **Very low quality:** We are very uncertain about the estimate. | | | | | | |
